# Etiologic Workup in Cases of Cryptogenic Stroke: A Systematic Review of International Clinical Practice Guidelines

**DOI:** 10.1161/STROKEAHA.119.027123

**Published:** 2020-04-13

**Authors:** Naoimh E. McMahon, Munirah Bangee, Valerio Benedetto, Emma P. Bray, Rachel F. Georgiou, Josephine M.E. Gibson, Deirdre A. Lane, A. Hakam Al-Khalidi, Kausik Chatterjee, Umesh Chauhan, Andrew J. Clegg, C. Elizabeth Lightbody, Gregory Y.H. Lip, Alakendu Sekhar, Caroline L. Watkins

**Affiliations:** 1 From the Faculty of Health and Wellbeing, University of Central Lancashire, Preston, United Kingdom (N.E.M., M.B., V.B., E.P.B., R.F.G., J.M.E.G., A.J.C., C.E.L., C.L.W.); 2 Liverpool Centre for Cardiovascular Science, University of Liverpool & Liverpool Heart and Chest Hospital, United Kingdom (D.A.L., G.Y.H.L.); 3 Department of Clinical Medicine, Aalborg University, Denmark (D.A.L., G.Y.H.L.); 4 Medtronic Limited, Watford, United Kingdom (A.H.A.-K.); 5 Countess of Chester Hospital, United Kingdom (K.C.); 6 Faculty of Clinical and Biomedical Sciences, University of Central Lancashire, Preston, United Kingdom (U.C.); 7 The Walton Centre NHS Foundation Trust, Liverpool, United Kingdom (A.S.).

**Keywords:** atrial fibrillation, diagnosis, neuroimaging, secondary prevention, stroke

## Abstract

Identifying the etiology of acute ischemic stroke is essential for effective secondary prevention. However, in at least one third of ischemic strokes, existing investigative protocols fail to determine the underlying cause. Establishing etiology is complicated by variation in clinical practice, often reflecting preferences of treating clinicians and variable availability of investigative techniques. In this review, we systematically assess the extent to which there exists consensus, disagreement, and gaps in clinical practice recommendations on etiologic workup in acute ischemic stroke.

**Methods—:**

We identified clinical practice guidelines/consensus statements through searches of 4 electronic databases and hand-searching of websites/reference lists. Two reviewers independently assessed reports for eligibility. We extracted data on report characteristics and recommendations relating to etiologic workup in acute ischemic stroke and in cases of cryptogenic stroke. Quality was assessed using the AGREE II tool (Appraisal of Guidelines for Research & Evaluation). Recommendations were synthesized according to a published algorithm for diagnostic evaluation in cryptogenic stroke.

**Results—:**

We retrieved 16 clinical practice guidelines and 7 consensus statements addressing acute stroke management (n=12), atrial fibrillation (n=5), imaging (n=5), and secondary prevention (n=1). Five reports were of overall high quality. For all patients, guidelines recommended routine brain imaging, noninvasive vascular imaging, a 12-lead ECG, and routine blood tests/laboratory investigations. Additionally, ECG monitoring (>24 hours) was recommended for patients with suspected embolic stroke and echocardiography for patients with suspected cardiac source. Three reports recommended investigations for rarer causes of stroke. None of the reports provided guidance on the extent of investigation needed before classifying a stroke as cryptogenic.

**Conclusions—:**

While consensus exists surrounding standard etiologic workup, there is little agreement on more advanced investigations for rarer causes of acute ischemic stroke. This gap in guidance, and in the underpinning evidence, demonstrates missed opportunities to better understand and protect against ongoing stroke risk.

**Registration—:**

URL: https://www.crd.york.ac.uk/PROSPERO/; Unique identifier: CRD42019127822.

## Abstract 2

Supplemental Digital Content is available in the text.

In at least one third of acute ischemic strokes, investigative protocols fail to establish the exact etiology.^1,2^ Etiologic workup in such cases of cryptogenic stroke, or stroke of unknown origin, is complicated by the varied emphasis of clinicians on establishing underlying cause because of the lack of evidence-based secondary prevention strategies^3–5^ and variable availability of different investigative techniques. Recent efforts to facilitate trials of secondary prevention strategies have resulted in the development of the embolic strokes of undetermined source construct (ESUS), which describes the subgroup of nonlacunar cryptogenic ischemic strokes in which embolism is considered the likely mechanism.^6^ Two trials of non–vitamin K antagonist oral anticoagulants in ESUS populations however failed to show a reduction in recurrent stroke when compared with aspirin,^7,8^ with one trial showing possible harm with an excess of bleeding.^7^ This may be because of unidentified heterogeneity even within the ESUS subgroup, resulting in the inclusion of patients who were unlikely to benefit from anticoagulation as a secondary prevention strategy.^9^ These trials have demonstrated that one size does not fit all and further highlighted the importance of systematic and evidence-based investigation of cryptogenic stroke to facilitate the development and implementation of personalized secondary prevention strategies. In this review, we use Saver’s^10^ algorithm for etiologic workup in cryptogenic stroke to systematically assess the extent to which there exists consensus, disagreement, and gaps in clinical practice recommendations on etiologic workup in acute ischemic stroke. The review findings highlight priorities for future research to inform more standardized approaches to evaluating cryptogenic stroke.

## Methods

The review was designed in accordance with the Preferred Reporting Items for Systematic Review and Meta-Analysis guidance. A Preferred Reporting Items for Systematic Review and Meta-Analysis checklist is provided in Appendix A in the Data Supplement. The protocol was prospectively registered on PROSPERO: CRD42019127822.

### Eligibility Criteria

Clinical practice guidelines (CPGs) were included if they (1) were endorsed by a national and/or international organization (eg, governmental, charitable, professional practice), (2) included recommendations about etiologic workup in acute ischemic stroke, (3) were published from January 2009 onwards (to ensure only the most up-to-date guidelines were included), and (4) were available in English. During our searches, we also identified scientific statements and consensus documents. Although these reports used less robust methods to search for and synthesize the underpinning evidence, the content was sufficiently relevant to the objectives of the review to merit inclusion. For transparency, recommendations from these publications are presented separately throughout.

### Search Strategy and Guideline Selection

We searched 4 electronic databases (MEDLINE, Health Management Information Consortium (HMIC), Embase, and CINAHL) from their inception to the 4th of March 2019 using a combination of Medical Subject Headings and keywords. As CPGs are often not indexed by electronic databases, we also systematically searched for guidelines on relevant websites including, but not limited to, the Guidelines International Network (www.g-i-n.net); The American Academy of Neurology (www.aan.com/); The World Stroke Organisation (www.world-stroke.org); and Open Grey (www.opengrey.eu/). Additionally, we reviewed the reference lists of included CPGs to identify relevant guidelines. The final list of CPGs/consensus statements was reviewed by all authors to confirm that no relevant documents, of which the team were already aware, had been omitted. The complete search strategy is provided in Appendix B in the Data Supplement. Two reviewers (Drs McMahon and Bangee) independently screened all retrieved citations for eligibility. Full texts of potentially relevant citations were obtained and independently assessed by both reviewers. Uncertainty was resolved through discussions with the review group.

### Data Collection and Quality Appraisal

A bespoke data extraction form was piloted before being finalized. For each guideline, one reviewer extracted all relevant information using this form, which was then fully checked by a second reviewer for completeness and accuracy. We extracted the following information: authors; organization; year of publication; country/region; development approach, evidence-assessment scales, and approach to producing recommendations; funding and disclosures; any content relating to the level of etiologic workup required in acute ischemic stroke. Relevant supplementary material cited in the guidelines was also retrieved and used to inform data extraction and quality appraisal.

The AGREE II tool (Appraisal of Guidelines for Research and Evaluation II^11^) was used to assess and illustrate the quality of the included publications. This tool includes 6 quality domains: scope and purpose; stakeholder involvement; rigor of development; clarity and presentation; applicability; and editorial independence. Each guideline was independently assessed by 4 appraisers from the review team (Dr McMahon, Dr Bangee, Dr Bray, Dr Gibson, R.F. Georgiou, Dr Benedetto, Dr Lane) and a quality score calculated for each domain as per the AGREE II formula.^12^ In line with similar reviews, we assessed agreement for each domain item and collectively reviewed items where appraisers scores were >1.5 SD from the mean item score.^13^ A domain was considered to be adequately addressed if scoring was ≥60%.^13–16^ The data extraction and quality appraisal forms are provided in Appendix C in the Data Supplement.

### Synthesis

All recommendations describing etiologic workup in acute ischemic stroke were collated in a spreadsheet and synthesized according to Saver’s^10^ algorithm for etiologic workup in cryptogenic stroke. Additional informal commentary was similarly collated in a spreadsheet and content analysis performed. These stages of refining and synthesizing the data were regularly discussed with the review team, particularly practising clinicians, to determine the consistency and appropriateness of the process and decision-making.

## Results

The electronic search strategy retrieved a total of 8442 citations. After the removal of duplicates and pre-2009 publications, 4566 were screened on title and abstract. We assessed 114 full texts for eligibility, of which 23 were included in the review (Figure). A full list of excluded records with reasons is provided in Appendix D in the Data Supplement.

**Figure.**
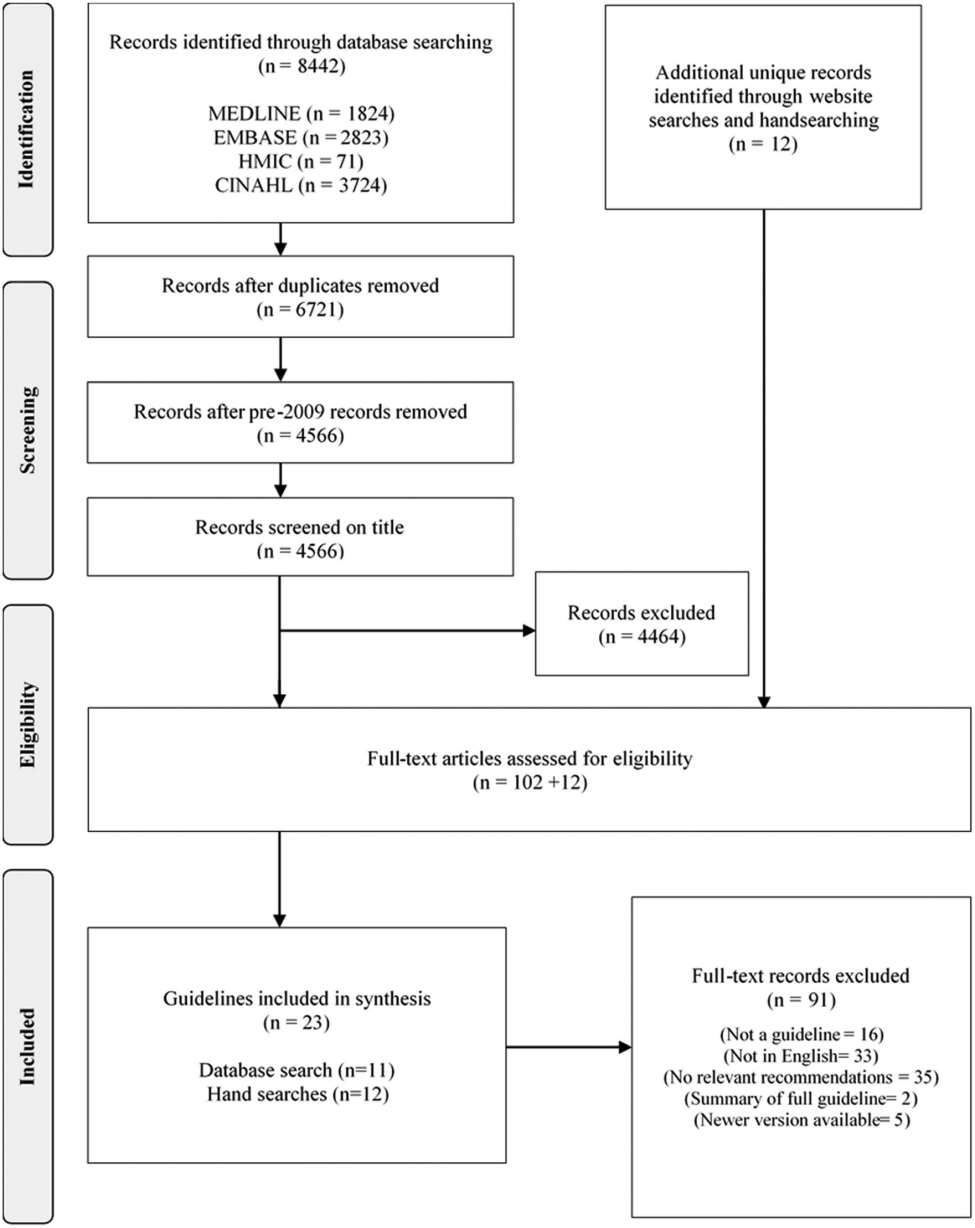
PRISMA flow diagram. HMIC indicates Health Management Information Consortium.

### Characteristics and Quality of the Included Guidelines/Statements

An overview of the included guidelines/statements is provided in Table 1.

**Table 1.**
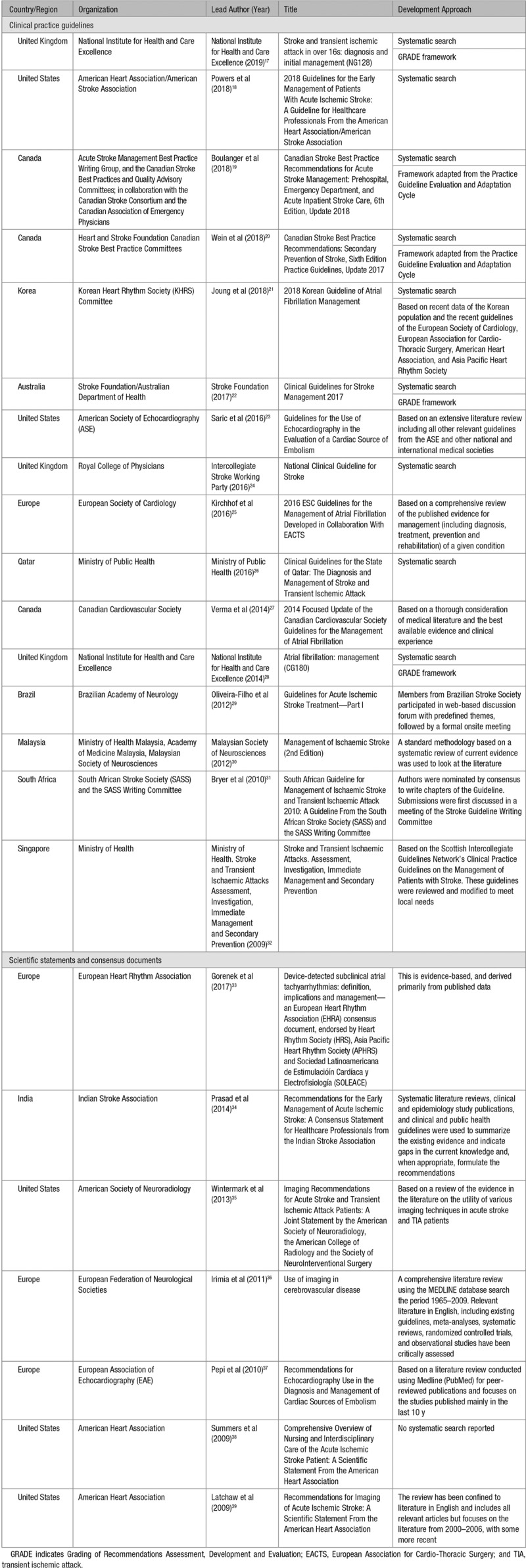
Characteristics of Included Guidelines and Consensus Statements

There were 16 CPGs^17–32^ and 7 organizational statements or consensus documents.^33–39^ Most publications came from American (n=5),^18,23,35,38,39^ European (n=3),^25,33,36,37^ Canadian (n=3),^19,20,27^ and British organizations (n=3).^17,24,28^ Topics included acute stroke management (n=12),^17–19,22,24,26,29–32,34,38^ atrial fibrillation and tachyarrhythmias (n=5),^21,25,27,28,33^ imaging in acute stroke (n=5),^23,35–37,39^ and secondary prevention (n=1).^20^ Just under half (n=11) were published from 2016 onwards.^17–26,33^ Two guidelines disclosed industry support in the production of the guidelines.^30,31^

The mean quality appraisal scores of 4 reviewers for each domain of the AGREE II are shown in Table 2, where green indicates domains which were adequately addressed (ie, ≥60%). For completeness, we also appraised the included consensus statements, which, as expected, scored less favorably than the CPGs. Almost all documents adequately addressed Domain 4 (clarity of presentation), which was the highest scoring domain followed by Domain 1 (scope and purpose). Applicability (Domain 5) scored most poorly, with this domain also noted to have the poorest agreement across raters. Five reports were of high quality overall, scoring ≥60% across all 5 domains.^17,19,20,22,28^

**Table 2.**
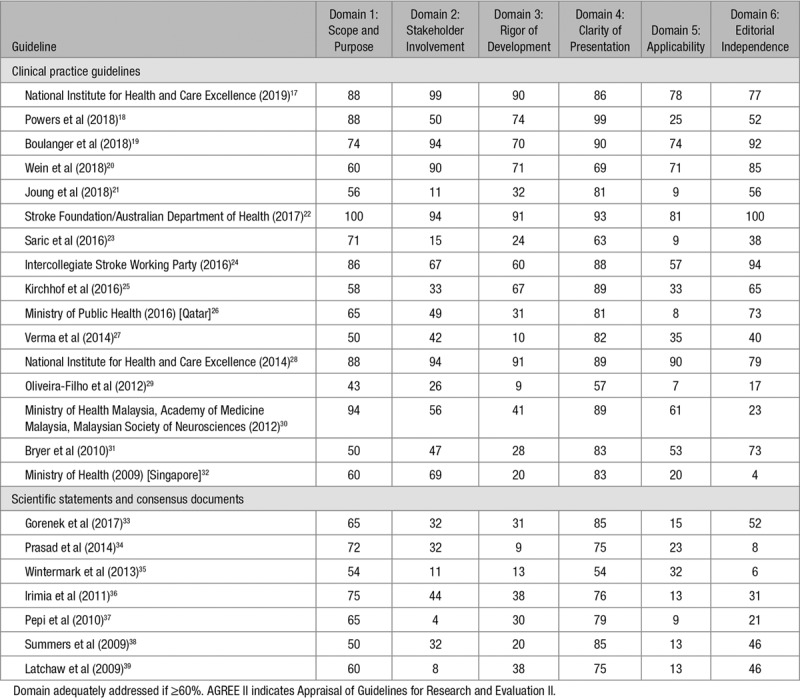
AGREE II Appraisal of Included Studies

### Establishing Stroke Etiology

Of the guidelines/statements specific to acute stroke management (n=12), 7 explicitly highlighted the importance of establishing stroke etiology^19,22,24,30–32,34^ (Table I in the Data Supplement). Two made recommendations on additional investigations to be performed for ESUS patients,^19,22^ with a further 4 reports providing recommendations on tests that should be considered in selected patients where cause has not been established through standard workup.^24,30,31,34^

Reflective of Saver’s algorithm,^10^ we organized recommendations into 6 categories of investigation: (1) brain imaging, (2) vascular imaging, (3) cardiac rhythm, (4) cardiac structure, (5) laboratory, and (6) other investigations (Table 3). Full details of all guideline recommendations with respect to these 6 categories of investigations are provided in Tables II through VII in the Data Supplement, while a summary of the recommendations for diagnostic workup in acute ischemic stroke can be found in Table 3. For reference purposes, the different evidence assessment scales and class of recommendations used in the included guidelines can also be found in Appendix E in the Data Supplement. For all patients with suspected acute stroke, guidelines recommend that they routinely undergo brain imaging, noninvasive vascular imaging, a 12-lead ECG, and routine blood tests/laboratory investigations. Recommendations on additional investigations included ECG monitoring for >24 hours for patients being investigated for embolic stroke (extended if atrial fibrillation is not detected but a cardioembolic source is suspected) and echocardiography for patients where etiology has not been established but a cardiac source is suspected. Three guidelines provided recommendations of further investigations for more unusual causes of stroke. These investigations included serology for Chagas disease and syphilis^29^ and, in younger people specifically, evaluation of autoimmune diseases, prothrombotic states (eg, antiphospholipid syndrome),^24,29^ Fabry disease,^24^ and thrombophilia.^26,29^

**Table 3.**
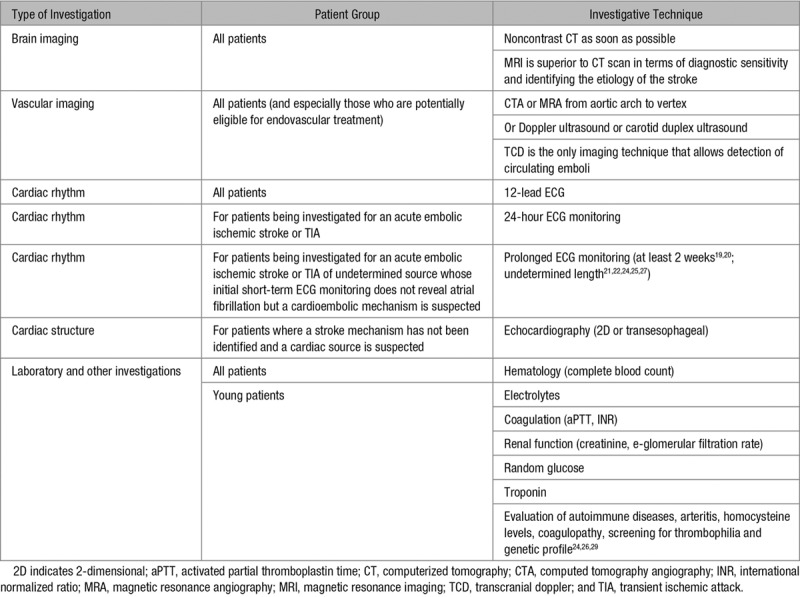
Overview of Recommendations for Diagnostic Workup in Acute Ischemic Stroke

While cryptogenic stroke was often discussed in the context of established classification systems (notably TOAST [Trial of ORG 10172 in Acute Stroke Treatment]^21,23,25–27,37^), none of the included guidelines/statements went beyond the TOAST categories to specifically identify when a stroke should be classified as cryptogenic (Tables VIII in the Data Supplement). More recently published guidelines using the ESUS construct included recommendations on prolonged cardiac monitoring, but lacked guidance on investigating other stroke mechanisms, and the extent to which investigations should be undertaken to establish stroke cause.

## Discussion

We have presented a systematic assessment of recommendations from international CPGs and consensus statements detailing etiologic workup in acute ischemic stroke. The review demonstrates that clear consensus exists on investigations which should be routinely performed for all acute ischemic stroke patients (standard evaluation^10^), but highlights the lack of consistency and detail on additional investigations for patients in whom a cause is not identified through standard evaluation. While recently published high-quality guidelines using the ESUS construct included recommendations for advanced evaluation focusing on prolonged ECG monitoring (ie, >24 hours), they do not yet provide guidance on the optimum or desired duration of monitoring. Indeed, the most recent update of the American Heart Association/American Stroke Association Guidelines for the Early Management of Acute Ischemic Stroke, published following the completion of this review, further reiterates that the effectiveness of prolonged cardiac monitoring for the purposes of guiding secondary prevention remains uncertain.^40^ Additionally, as ESUS represents only a subgroup of cryptogenic stroke, guidance is still lacking for those patients where the stroke mechanism is not embolic. Indeed, consideration of more unusual causes of stroke was limited to just 3 reports, all of which were published in 2016 or earlier.^24,26,29^ It was therefore not possible to identify a standardized evaluation approach from current guidelines, suggesting that practice variability in investigating cryptogenic stroke is inevitable. Practice variability is likely to be further compounded by the limited attention paid to the applicability of recommendations across included reports, a limitation of CPGs often highlighted in published reviews.^13,14^

This review has highlighted the need for well-designed primary research to identify an optimal pathway to expedite the identification of rare and very rare stroke etiologies in a timely and cost-effective manner. A significant challenge to further clinically based research is, however, the rarity of these causes. Additionally, as treating healthcare professionals are deeply engaged in dealing with the consequences of the current stroke, advanced etiologic workup often takes a back seat. While the TOAST classification acts as a useful starting point, it is evident that further research is needed to underpin and guide investigation in clinical practice. However, because of the lack of individualized secondary prevention strategies, such research should include economic analysis to compare the costs, risks, and benefits of less or more exhaustive approaches, while also exploring variation in stroke subtype by race and ethnicity, along with genetic differences. Importantly, the perspectives of stroke survivors and family members are paramount and should guide future research and implementation, enabling a personalized approach for each individual based not only on their clinical presentation but also on their values, needs, and preferences.

## Conclusions

Current CPGs on the etiologic workup of acute ischemic stroke are of variable quality but largely reach consensus about appropriate standard investigations. There is, however, little agreement and a lack of underpinning evidence for more advanced or specialized investigations for rarer causes of stroke. This lack of evidence and consensus, along with poor applicability of many of the existing guidelines, is likely to contribute to variability of access to investigations, inappropriate use of costly and specialized resources and skills, along with delays or lack of diagnosis of etiologies. Unless addressed, this gap in knowledge will continue to result in missed opportunities to identify and implement necessary secondary prevention measures and provide high-quality clinical and psychological advice and support to stroke survivors and their families in relation to ongoing stroke risk.

## Sources of Funding

This work is funded by a Liverpool Clinical Commissioning Group (LCCG) Research Capability Funding grant (Investigating the Detection of Cryptogenic Stroke [ID-CRYPT]) awarded to Professor Dame Caroline Watkins. Drs Watkins, McMahon, Benedetto, and Clegg are part-funded by the National Institute for Health Research Applied Research Collaboration North West Coast (NIHR ARC NWC). The views expressed are those of the author(s) and not necessarily those of the NIHR or the Department of Health and Social Care.

## Disclosures

Dr Lane has received investigator-initiated educational grants from Bristol-Myers Squibb and Boehringer Ingelheim; has been a speaker for Boehringer Ingelheim, Bayer, and Bristol-Myers Squibb/Pfizer; and has consulted for Bristol-Myers Squibb, Bayer, Boehringer Ingelheim, and Daiichi-Sankyo. Dr Lip has Consulted for Bayer/Janssen, BMS/Pfizer, Medtronic, Boehringer Ingelheim, Novartis, Verseon, and Daiichi-Sankyo and has been a speaker for Bayer, BMS/Pfizer, Medtronic, Boehringer Ingelheim, and Daiichi-Sankyo. No fees are directly received personally. Dr Al-Khalidi receives a salary from Medtronic Ltd, United Kingdom. The other authors report no conflicts.

## Supplementary Material


