# Supplementary material for: Etiologic Workup in Cases of Cryptogenic Stroke: A Systematic Review of International Clinical Practice Guidelines
Source: Stroke. 2020 Apr 13;51(5):1419–27. doi: 10.1161/STROKEAHA.119.027123 (PMC7185056; doi:10.1161/STROKEAHA.119.027123)
Supplement: Supplementary file 1 [file str-51-1419-s001.pdf]

SUPPLEMENTAL MATERIAL

Abbreviations 2
Appendix A. PRISMA Checklist 3
Appendix B. Complete search strategy 6
Appendix C. Data extraction and quality appraisal forms 8
Appendix D. Excluded citations with reasons 14
Appendix E. Evidence assessment scales 23
Table I. Commentary in the stroke management guidelines/statements on establishing stroke etiology 36
Table II. Recommendations for brain imaging 42
Table III. Recommendations for vascular imaging 46
Table IV. Recommendations for ECG monitoring 51
Table V. Recommendations for investigations of cardiac structure 56
Table VI. Recommendations for laboratory investigations 60
Table VII. Recommendations for other investigations 62
Table VIII. Commentary in the guidelines/statements relating to when a stroke should be classified as cryptogenic 64

Abbreviations

AF	Atrial Fibrillation
aPTT	Activated Partial Thromboplastin Time
AVM	Arteriovenous Malformation
CBC	Complete Blood Count
CT	Computed Tomography
CTA	Computed Tomography Angiography/Angiogram
DSA	Digital Subtraction Angiography
DWI	Diffusion-Weighted Imaging
ECG	Electrocardiogram
ED	Emergency Department
INR	International Normalized Ratio
MAC	Mitral Annulus Calcification
MVP	Mitral Valve Prolapse
MRA	Magnetic Resonance Angiography/Angiogram
MRI	Magnetic Resonance Imaging
OAC	Oral Anticoagulation
PFO	Patent Foramen Ovale
PT	Prothrombin Time
rtPA	Recombinant Tissue Plasminogen Activator
TCD	Transcranial Doppler
TEE/TOE	Transesophageal Echocardiography
TIA	Transient Ischaemic Attack

Appendix A. PRISMA Checklist

Section/topic	#	Checklist item	Reported on page #
**TITLE**			
Title	1	Identify the report as a systematic review, meta-analysis, or both.	1
**ABSTRACT**			
Structured summary	2	Provide a structured summary including, as applicable: background; objectives; data sources; study eligibility criteria, participants, and interventions; study appraisal and synthesis methods; results; limitations; conclusions and implications of key findings; systematic review registration number.	32-62
**INTRODUCTION**			
Rationale	3	Describe the rationale for the review in the context of what is already known.	67-83
Objectives	4	Provide an explicit statement of questions being addressed with reference to participants, interventions, comparisons, outcomes, and study design (PICOS).	83-88
**METHODS**			
Protocol and registration	5	Indicate if a review protocol exists, if and where it can be accessed (e.g., Web address), and, if available, provide registration information including registration number.	91-93
Eligibility criteria	6	Specify study characteristics (e.g., PICOS, length of follow-up) and report characteristics (e.g., years considered, language, publication status) used as criteria for eligibility, giving rationale.	94-103
Information sources	7	Describe all information sources (e.g., databases with dates of coverage, contact with study authors to identify additional studies) in the search and date last searched.	104-115
Search	8	Present full electronic search strategy for at least one database, including any limits used, such that it could be repeated.	Appendix B
Study selection	9	State the process for selecting studies (i.e., screening, eligibility, included in systematic review, and, if applicable, included in the meta-analysis).	115-118

Data collection process	10	Describe method of data extraction from reports (e.g., piloted forms, independently, in duplicate) and any processes for obtaining and confirming data from investigators.	119-138
Data items	11	List and define all variables for which data were sought (e.g., PICOS, funding sources) and any assumptions and simplifications made.	122-126
Risk of bias in individual studies	12	Describe methods used for assessing risk of bias of individual studies (including specification of whether this was done at the study or outcome level), and how this information is to be used in any data synthesis.	128-138
Summary measures	13	State the principal summary measures (e.g., risk ratio, difference in means).	n/a
Synthesis of results	14	Describe the methods of handling data and combining results of studies, if done, including measures of consistency (e.g., I^2) for each meta-analysis.	n/a
Risk of bias across studies	15	Specify any assessment of risk of bias that may affect the cumulative evidence (e.g., publication bias, selective reporting within studies).	n/a
Additional analyses	16	Describe methods of additional analyses (e.g., sensitivity or subgroup analyses, meta-regression), if done, indicating which were pre-specified.	n/a
**RESULTS**			
Study selection	17	Give numbers of studies screened, assessed for eligibility, and included in the review, with reasons for exclusions at each stage, ideally with a flow diagram.	147-151
Study characteristics	18	For each study, present characteristics for which data were extracted (e.g., study size, PICOS, follow-up period) and provide the citations.	Table 1<br>153-161
Risk of bias within studies	19	Present data on risk of bias of each study and, if available, any outcome level assessment (see item 12).	n/a
Results of individual studies	20	For all outcomes considered (benefits or harms), present, for each study: (a) simple summary data for each intervention group (b) effect estimates and confidence intervals, ideally with a forest plot.	Tables II – VIII in appendix
Synthesis of results	21	Present results of each meta-analysis done, including confidence intervals and measures of consistency.	172-202
Risk of bias across studies	22	Present results of any assessment of risk of bias across studies (see Item 15).	Table 2<br>163-170

Additional analysis	23	Give results of additional analyses, if done (e.g., sensitivity or subgroup analyses, meta-regression [see Item 16]).	n/a
**DISCUSSION**			
Summary of evidence	24	Summarize the main findings including the strength of evidence for each main outcome; consider their relevance to key groups (e.g., healthcare providers, users, and policy makers).	203-233
Limitations	25	Discuss limitations at study and outcome level (e.g., risk of bias), and at review-level (e.g., incomplete retrieval of identified research, reporting bias).	215-217
Conclusions	26	Provide a general interpretation of the results in the context of other evidence, and implications for future research.	234-244
**FUNDING**			
Funding	27	Describe sources of funding for the systematic review and other support (e.g., supply of data); role of funders for the systematic review.	245-253

From: Moher D, Liberati A, Tetzlaff J, Altman DG, The PRISMA Group (2009). Preferred Reporting Items for Systematic Reviews and Meta-Analyses: The PRISMA Statement. PLoS Med 6(7): e1000097. doi:10.1371/journal.pmed1000097

For more information, visit: **www.prisma-statement.org**.

Appendix B. Complete search strategy

Electronic search strategy (searches run 4th March 2019)

MEDLINE (Ovid MEDLINE(R) and Epub Ahead of Print, In-Process & Other Non-Indexed Citations)

1	exp Guideline/	31558
2	exp Practice Guideline/	24799
3	(guideline or guidelines).ti	69905
4	1 or 2 or 3	88328
5	exp Stroke/	119932
6	Stroke.mp	262121
7	5 or 6	282813
8	4 and 7	1824

HMIC Health Management Information Consortium

1	exp Guidelines/	6789
2	exp Clinical guidelines/	1485
3	(guideline or guidelines).ti	3140
4	1 or 2 or 3	8427
5	exp Stroke/	1702
6	Stroke.mp	2637
7	6 or 7	2652
8	4 and 7	71

Embase

1	(guideline or guidelines).ti	91859
2	exp cerebrovascular accident/	181373
3	Stroke.mp	397857
4	2 or 3	447483
5	1 and 4	2823

CINAHL Complete

S1	(MH “Practice Guidelines”)	66593
S2	TI guideline or guidelines	36845
S3	TX stroke	187617
S4	(MH “Stroke+)	59532
S5	S1 or S2	85546
S6	S3 or S4	187617
S7	S5 and S6	3724

Hand-searching of websites and via search engines (searches run 18th Feb 2019)

Website	Address	All hits exported for screening
Guidelines International Network	https://www.g-i-n.net/	92
Scottish Intercollegiate Guidelines Network	https://www.sign.ac.uk/	2
National Institute for Health and Care Excellence	https://www.nice.org.uk/	41
Stroke Foundation	https://informme.org.au	4
Royal College of Physicians	https://www.rcplondon.ac.uk	14
American Academy of Neurology	https://www.aan.com/	50
European Stroke Organisation	https://eso-stroke.org/eso-guideline-directory/	23
World Stroke Organisation document	https://www.world-stroke.org/2016-12-19-10-55-24/clinical-practice-guideline	71
Google	https://www.google.com/	n/a

Appendix C. Data extraction and quality appraisal forms

1. Data Extraction

Guideline characteristics	
Guideline name:	
Organisation(s):	
Country:	
Type of guideline/document:	
Publication date:	
Planned review/update date:	
Relevant objectives (i.e. related to investigation):	
Target audience:	
Development process (i.e. general approach):	
Systematic search processes:	
Process for linking levels of evidence to grades of recommendations:	
Funder:	
Role of the funder:	
Link to guideline:	
Any related or supplementary material/documents:	
Documents used for data extraction:	

Admin	
Reviewer 1:	
Date completed by Reviewer 1:	
Reviewer 2:	
Date checks completed by Reviewer 2:	
Name and location of final saved DE form:	

Levels of evidence	Grade of recommendations

Cryptogenic stroke	Yes/No - provide relevant text
Explicitly mentions cryptogenic stroke or stroke of unknown source	
Uses other terms to describe cryptogenic stroke or stroke of unknown source	
Provides details on defining or classifying cryptogenic stroke or stroke of unknown source	

Formal recommendations related to investigation and classification of seemingly cryptogenic stroke	

FORMAL recommendations related to assessment, investigation and diagnosis of stroke aetiology

Section of the guideline and page number	Recommendation	Grade	Evidence cited to support grade	Notes

INFORMAL commentary related to assessment, investigation and diagnosis of stroke aetiology

Section of the guideline and page number	Recommendation	Notes

2. Quality appraisal

Admin
Assessor:
Documents used in the assessment:
Data appraisal fully completed:

Quality Appraisal		
**Domain 1: Scope and purpose**	**Score**	**Notes**
1. The overall objective(s) of the guideline is (are) specifically described.		
2. The health question(s) covered by the guideline is (are) specifically described.		
3. The population (patients, public, etc.) to whom the guideline is meant to apply is specifically described.		
**Domain 2: Stakeholder involvement**	**Score**	**Notes**
4. The guideline development group includes individuals from all relevant professional groups.		
5. The views and preferences of the target population (patients, public, etc.) have been sought.		
6. The target users of the guideline are clearly defined.		
**Domain 3: Rigour of development**	**Score**	**Notes**
7. Systematic methods were used to search for evidence.		
8. The criteria for selecting the evidence are clearly described.		
9. The strengths and limitations of the body of evidence are clearly described.		
10. The methods for formulating the recommendations are clearly described.		
11. The health benefits, side effects, and risks have been considered in formulating the recommendations.		
12. There is an explicit link between the recommendations and the supporting evidence.		
13. The guideline has been externally reviewed by experts prior to its publication.		
14. A procedure for updating the guideline is provided.		
**Domain 4: Clarity of presentation**	**Score**	**Notes**
15. The recommendations are specific and unambiguous.		
16. The different options for management of the condition or health issue are clearly presented.		
17. Key recommendations are easily identifiable.		

Domain 5: Applicability	Score	Notes
18. The guideline describes facilitators and barriers to its application.		
19. The guideline provides advice and/or tools on how the recommendations can be put into practice.		
20. The potential resource implications of applying the recommendations have been considered.		
21. The guideline presents monitoring and/or auditing criteria.		
**Domain 6: Editorial independence**	**Score**	**Notes**
22. The views of the funding body have not influenced the content of the guideline.		
23. Competing interests of guideline development group members have been recorded and addressed.		
**Overall guideline assessment**	**Score**	**Notes**
1. Rate the overall quality of this guideline.		
2. I would recommend this guideline for use.		

3. Collating quality appraisal scores across four independent raters

Quality Appraisal							
**Domain 1: Scope and purpose**	**QA 1**	**QA 2**	**QA 3**	**QA 4**	**Total**	**Means**	**Domain score (%)**
1. The overall objective(s) of the guideline is (are) specifically described.							
2. The health question(s) covered by the guideline is (are) specifically described.							
3. The population (patients, public, etc.) to whom the guideline is meant to apply is specifically described.							
**Totals**							
**Domain 2: Stakeholder involvement**	**QA 1**	**QA 2**	**QA 3**	**QA 4**	**Total**	**Means**	**Domain score (%)**
4. The guideline development group includes individuals from all relevant professional groups.							
5. The views and preferences of the target population (patients, public, etc.) have been sought.							
6. The target users of the guideline are clearly defined.							
**Totals**							
**Domain 3: Rigour of development**	**QA 1**	**QA 2**	**QA 3**	**QA 4**	**Total**	**Means**	**Domain score (%)**
7. Systematic methods were used to search for evidence.							
8. The criteria for selecting the evidence are clearly described.							
9. The strengths and limitations of the body of evidence are clearly described.							
10. The methods for formulating the recommendations are clearly described.							
11. The health benefits, side effects, and risks have been considered in formulating the recommendations.							
12. There is an explicit link between the recommendations and the supporting evidence.							
13. The guideline has been externally reviewed by experts prior to its publication.							
14. A procedure for updating the guideline is provided.							
**Totals**							
**Domain 4: Clarity of presentation**	**QA 1**	**QA 2**	**QA 3**	**QA 4**	**Total**	**Means**	**Domain score (%)**
15. The recommendations are specific and unambiguous.							
16. The different options for management of the condition or health issue are clearly presented.							

17. Key recommendations are easily identifiable.							
**Totals**							
**Domain 5: Applicability**	**QA 1**	**QA 2**	**QA 3**	**QA 4**	**Total**	**Means**	**Domain score (%)**
18. The guideline describes facilitators and barriers to its application.							
19. The guideline provides advice and/or tools on how the recommendations can be put into practice.							
20. The potential resource implications of applying the recommendations have been considered.							
21. The guideline presents monitoring and/or auditing criteria.							
**Totals**							
**Domain 6: Editorial independence**	**QA 1**	**QA 2**	**QA 3**	**QA 4**	**Total**	**Means**	**Domain score (%)**
22. The views of the funding body have not influenced the content of the guideline.							
23. Competing interests of guideline development group members have been recorded and addressed.							
**Totals**							
**Overall guideline assessment**	**QA 1**	**QA 2**	**QA 3**	**QA 4**			
1. Rate the overall quality of this guideline.							
2. I would recommend this guideline for use.							

Appendix D. Excluded citations with reasons

Not a national clinical guideline (n=16)

1. Bae HJ. Clinical practice guideline for stroke in Korea. Clin Neurol. 2010;50:825.
2. Brazzelli MG, Sandercock P, Wardlaw J. Evidence-based guideline: the role of diffusion and perfusion MRI for the diagnosis of acute ischemic stroke: report of the Therapeutics and Technology Subcommittee of the American Academy of Neurology. Neurol. 2011;76:2036-2037.
3. Brieger D, Amerena J, Attia JR, Bajorek B, Chan KH, Connell C, et al. National Heart Foundation of Australia and Cardiac Society of Australia and New Zealand: Australian clinical guidelines for the diagnosis and management of atrial fibrillation 2018. Med J Aust. 2018;209:356-362.
4. Fisher M. Update on the Early Management of Patients With Acute Ischemic Stroke Guidelines. Stroke. 2019;50:2.
5. Furie KL, Jayaraman MV. 2018 Guidelines for the Early Management of Patients With Acute Ischemic Stroke. Stroke. 2018;49:509-510.
6. Hobbs FR, Taylor CJ, Jan Geersing G, Rutten FH, Brouwer JR, European Primary Care Cardiovascular Society (EPCCS) SPAF working group. European Primary Care Cardiovascular Society (EPCCS) consensus guidance on stroke prevention in atrial fibrillation (SPAF) in primary care. Eur J Prev Cardiol. 2016;23:460-473.
7. Kes VB, Cesarik M, Zavoreo I, Soldo-Butkovic S, Kes P, Basic-Jukic N, et al. Guidelines for diagnosis, therapy and follow up of anderson-fabry disease. Acta Clin Croat. 2013;52:395-405.
8. Lahri S, Wallis L. South African ischaemic stroke guideline, 2010. S Afr Med J. 2011;101:7.
9. Linden B. ESC clinical guideline on management of atrial fibrillation. Br J Card Nurs. 2011;6:240-242.
10. Lip GYH, Collet JP, Caterina R, Fauchier L, Lane DA, Larsen TB, et al. Antithrombotic therapy in atrial fibrillation associated with valvular heart disease: a joint consensus document from the European Heart Rhythm Association (EHRA) and European Society of Cardiology Working Group on Thrombosis, endorsed by the ESC Working Group on Valvular Heart Disease, Cardiac Arrhythmia Society of Southern Africa (CASSA), Heart Rhythm Society (HRS), Asia Pacific Heart Rhythm Society (APHRS), South African Heart (SA Heart) Association and Sociedad Latinoamericana de Estimulacion Cardiaca y Electrofisiologia (SOLEACE). Europace. 2017;19:1757-1758.
11. Saver JL, Wasiak H, Saver JL, Wasiak H. Stroke Council and American Stroke Association update. Stroke. 2011;42:830-831.
12. Scheinfeld MH, Krieger DA, Bhupali D, Zampolin RL, Erdfarb AJ. Imaging Scales and Techniques Used in the 2015 Endovascular Stroke Trials and AHA/ASA Revised Guidelines for Acute Intervention: Neurologic/Head and Neck Imaging. RadioGraphics. 2017;37:1605-1606.

13. Schwartz R, Schilling R. ESC guidelines on managing AF. Prescriber. 2013;24:7.

14. Steiner T, Al-Shahi Salman R, Ntaios G. The European Stroke Organisation (ESO) guidelines. Int J Stroke. 2014;9:838-839.

15. Uehara T, Minematsu K. Guidelines for management of patients with transient ischemic attack. Front Neurol Neurosci. 2014;33:103-114.

16. Yan-sheng LI. New ideas and inspiration of the updated guidelines for secondary prevention of ischemic stroke. Chin J Contemp Neurol Neurosurg. 2015;15:182-186.

Not in English (n=33)

1. Alonso de Lecinana M, Egido JA, Casado I, Ribo M, Davalos A, Masjuan J, et al. Guia para el tratamiento del infarto cerebral agudo. [Guidelines for the treatment of acute ischaemic stroke.] Neurologia. 2014;29:102-122.

2. Arsava EM, Ozturk V, Kutluk K, Uzuner N. Iskemik inme tanisi: turk beyin damar hastaliklari dernegi inme Tani Ve Tedavi kilavuzu - 2015. [Diagnosis of ischemic stroke: Guidelines of Turkish society of cerebrovascular diseases - 2015.] Turk Beyin Damar Hast Derg. 2015;21:80-84.

3. Bakar M, Ozdag MF, Melek I, Uluduz D, Uzuner GT, Armagan O, et al. Inme sonrasi: turk beyin damar hastaliklari dernegi inme tani ve tedavi kilavuzu 2015. [After stroke: Guidelines of Turkish Society of cerebrovascular diseases 2015.] Turk Beyin Damar Hast Derg. 2015;21:169-179.

4. Barzel A, Hensler S, Koneczny N, Muche-Borowski C, Scherer M. Aktualisierte DEGAM-leitlinie Schlaganfall - Was ist neu?. [Updated DEGAM guideline stroke - What's new?.] Z Allgemeinmed. 2014;90:152-157.

5. Castano-Guerra RJ, Franco-Vergara BC, Baca-Lopez FM, Aviles-Valverde J, Gonzalez-Aceves EN, Gonzalez-Hermosillo JA, et al. Guia de practica clinica. Diagnostico y tratamiento de la fibrilacion auricular. [Clinical guideline for diagnosis and treatment of atrial fibrillation]. Rev Med Inst Mex Seguro Soc. 2012;50:213-231.

6. Chinese Society of Cerebral Blood F, Metabolism. [The Chinese guidelines for the evaluation and management of cerebral collateral circulation in ischemic stroke (2017)]. Chung Hua Nei Ko Tsa Chih. 2017;56:460-471.

7. Czlonkowska A, Kobayashi A. Postepowanie w udarze mozgu - wytyczne Grupy Ekspertow Sekcji Chorob Naczyniowych Polskiego Towarzystwa Neurologicznego. Aktualizacja 2013: leczenie trombolityczne. [Management of acute stroke - guidelines from the Expert Group of the Section of Cerebrovascular Diseases of the Polish Neurological Society. Update 2013: thrombolysis]. Neurol Neurochir Pol. 2013;47:303-39.

8. Damen-van Beek Z. NHG-Standaard 'Beroerte'. [The guideline 'Stroke' of the Dutch College of General Practitioners.] Geneesmiddelenbulletin. 2014;48:49-50.

9. Davoodi R, Ghandehari K, Ghayeni MR, Harirchian MH, Vahdat HB, Nezhad S, et al. [Developing a national guideline for diagnosis, treatment and follow-up of stroke.] Journal of Mazandaran University of Medical Sciences. 2018;28:1-20.

10. Diener HC, Grau AJ, Baldus S, Ghanem A, Groschel K, Liebetrau C, et al. Kryptogener Schlaganfall und offenes Foramen ovale: S2e-Leitlinie. [Cryptogenic stroke and patent foramen ovale: S2e guidelines.] Nervenarzt. 2018;89:1143-1153.

11. Fuentes B, Gallego J, Gil-Nunez A, Morales A, Purroy F, Roquer J, et al. Guia para el tratamiento preventivo del ictus isquemico y AIT (I). Actuacion sobre los factores de riesgo y estilo de vida. [Guidelines for the preventive treatment of ischaemic stroke and TIA (I). Update on risk factors and life style.]. Neurologia. 2012;27:560-574.

12. Fuentes B, Gallego J, Gil-Nunez A, Morales A, Purroy F, Roquer J, et al. Guia para el tratamiento preventivo del ictus isquemico y AIT (II). Recomendaciones segun subtipo etiologico. [Guidelines for the preventive treatment of ischaemic stroke and TIA (II). Recommendations according to aetiological sub-type.] Neurologia. 2014;29:168-183.

13. Hu X, Wester P, Stibrant Sunnerhaagen K. Evidensbaserad rehabilitering efter stroke med nya riktlinjer. [Evidence-based methods in the clinical practice in updated Swedish national stroke guidelines.] Lakartidningen. 2018;115.

14. Jeng JS, Liu HM, Lee TH, Chang FC, Tu YK, Chiu HC, et al. [Guidelines for the management of carotid artery stenosis: a statement from Taiwan Stroke Society Guideline Committee of Carotid Artery Stenosis Management]. Acta Neurol Taiwan. 2009;18:64-76.

15. Kutluk K, Balkan S, Coskun O, Melek I, Gungor L, Dora B, et al. Ikincil koruma: turk beyin damar hastaliklari dernegi inme tani ve tedavi kilavuzu 2015. [Secondary prevention: Guidelines of Turkish Society of cerebrovascular diseases 2015.] Turk Beyin Damar Hast Derg. 2015;21:161-168.

16. Laufs U, Hoppe UC, Rosenkranz S, Kirchhof P, Bohm M, Diener HC, et al. [Cardiac workup after cerebral ischemia. Consensus paper of the Working Group on Heart and Brain of the German Cardiac Society and German Stroke Society]. Nervenarzt. 2010;81:444-462.

17. Lefaucheur JP, Andre-Obadia N, Poulet E, Devanne H, Haffen E, Londero A, et al. Recommandations francaises sur l'utilisation de la stimulation magnetique transcranienne repetitive (rTMS): Regles de securite et indications therapeutiques. [French guidelines on the use of repetitive transcranial magnetic stimulation (rTMS): Safety and therapeutic indications.] Neurophysiol Clin. 2011;41:221-295.

18. Martins SCO, Freitas GR, Pontes-Neto OM, Pieri A, Moro CHC, Jesus PAP, et al. Diretrizes para o tratamento do acidente vascular cerebral isquemico - parte ii: Tratamento do acidente vascular. [Guidelines for acute ischemic stroke treatment - part II: Stroke treatment.] Arq Neuropsiquiatr. 2012;70:885-893.

19. Melon P, Lancellotti P. Recommandations Europeennes 2010 pour le traitement anti-thrombotique de la fibrillation auriculaire: Nouveaux scores pour l'evaluation des risques d'accident vasculaire cerebral et de saignement. [2010 European guidelines for antithrombotic therapy in patients with atrial fibrillation.] Rev Med Liege. 2010;65:580-582.

20. Nazliel B, Gungor L, Topcuoglu MA, Tolun R, Ozturk V, Kutluk K, et al. Inme hastalarina ilk mudahale: turk beyin damar hastaliklari dernegi inme Tani Ve Tedavi kilavuzu - 2015. [Early management of patients with acute stroke: Guidelines of

Turkish society of cerebrovascular diseases - 2015.] Turk Beyin Damar Hast Derg. 2015;21:68-73.

21. Overbeck P, Europaische Gesellschaft fur K. [Update 2012: new European guidelines for atrial fibrillation]. MMW Fortschr Med. 2012;154:18-19.

22. Ozdemir AO, Yaka E, Tolun R, Giray S, Gungor L, Kutluk K, et al. Ozel iskemik inme tedavisi: turk beyin damar hastaliklari dernegi inme Tani Ve Tedavi kilavuzu - 2015. [Specific management of ischemic stroke: Guidelines of Turkish society of cerebrovascular diseases - 2015.] Turk Beyin Damar Hast Derg. 2015;21:93-98.

23. Raviele A, Disertori M, Alboni P, Bertaglia E, Botto G, Brignole M, et al. [2010 AIAC Guidelines for the management and treatment of atrial fibrillation]. G Ital Cardiol. 2011;12:7-69.

24. Rigau Comas D, Alvarez-Sabin J, Gil Nunez A, Abilleira Castells S, Borras Perez FX, Armario Garcia P, et al. Guia de practica clinica sobre prevencion primaria y secundaria del ictus. [Primary and secondary prevention of stroke: A guideline.] Med Clin. 2009;133:754-762.

25. Ringleb PA, Hamann GF, Rother J, Jansen O, Groden C, Veltkamp R. Akuttherapie des ischamischen Schlaganfalls - Rekanalisierende Therapie: Erganzung 2015. [Therapy of Acute Ischemic Stroke - Recanalisation Therapy Guideline: Update 2015.] Aktuelle Neurol. 2016;43:82-91.

26. Rivera-Nava SC, Miranda-Medrano LI, Perez-Rojas JE, de Jesus Flores J, Rivera-Garcia BE, del Pilar Torres-Arreola L. Guia de practica clinica. Enfermedad vascular cerebral isquemica. [Clinical guideline for the prevention, diagnosis and treatment of ischemic cerebral disease]. Rev Med Inst Mex Seguro Soc. 2012;50:335-346.

27. Schellinger PD, Bryan RN, Caplan LR. Evidence-based guideline: The role of diffusion and perfusion MRI for the diagnosis of acute ischemic stroke. [Chinese]. Chinese Journal of Cerebrovascular Diseases. 2010;7:668-672.

28. Shinohara Y. Japanese guidelines for the management of stroke 2009 - Important revised points necessary for the neurologist. [Japanese]. Clin Neurol. 2010;50:808-811.

29. Shinohara Y. [The cutting-edge of medicine: Japanese guidelines for the management of stroke 2009--the way to utilize and cardinal points for primary and secondary stroke prevention for internists]. Nippon Naika Gakkai Zasshi. 2011;100:497-502.

30. Skoda O, Herzig R, Mikulik R, Neumann J, Vaclavik D, Bar M, et al. Klinicky standard pro diagnostiku a lecbu pacientu s ischemickou cevni mozkovou prihodou a s tranzitorni ischemickou atakou - verze 2016. [Clinical guideline for the diagnostics and treatment of patients with ischemic stroke and transitory ischemic attack - Version 2016.] Ceska a Slovenska Neurologie a Neurochirurgie. 2016;79:351-363.

31. Suomalaisen Laakariseuran D, Suomen Neurologinen Yhdistys ryn Asettama T. Kaypa hoito -suosituksen padivitystiivistelma: aivoinfarkti. [Update on current care guidelines: cerebral infarction (stroke)]. Duodecim. 2011;127:500-501.

32. Topcuoglu MA, Utku U, Ince B, Ozdemir AO, Bas DF, Kutluk K, et al. Inme unitesinde genel inme tedavisi: turk beyin damar hastaliklari dernegi inme Tani Ve Tedavi kilavuzu - 2015. [General stroke management in stroke unit: Guidelines of

Turkish society of cerebrovascular diseases - 2015.] Turk Beyin Damar Hast Derg. 2015;21:89-92.

33. Uchiyama S. Japanese guidelines for the management of stroke 2009. Nihon Ronen Igakkai Zasshi. 2011;48:633-636.

No relevant recommendations (n=35)

1. Brieger D, Amerena J, Attia J, Bajorek B, Chan KH, Connell C, et al. National Heart Foundation of Australia and the Cardiac Society of Australia and New Zealand: Australian Clinical Guidelines for the Diagnosis and Management of Atrial Fibrillation 2018. Heart Lung Circ. 2018;27:1209-1266.

2. Andrade JG, Verma A, Mitchell LB, Parkash R, Leblanc K, Atzema C, et al. 2018 Focused Update of the Canadian Cardiovascular Society Guidelines for the Management of Atrial Fibrillation. Can J Cardiol. 2018;34:1371-1392.

3. Blacquiere D, Lindsay MP, Foley N, Taralson C, Alcock S, Balg C, et al. Canadian Stroke Best Practice Recommendations: Telestroke Best Practice Guidelines Update 2017. Int J Stroke. 2017;12:886-895.

4. Cairns JA, Connolly S, McMurtry S, Stephenson M, Talajic M. Canadian Cardiovascular Society atrial fibrillation guidelines 2010: Prevention of stroke and systemic thromboembolism in atrial fibrillation and flutter. Can J Cardiol. 2011;27:74-90.

5. Camm AJ, Lip GY, De Caterina R, Savelieva I, Atar D, Hohnloser SH, et al. 2012 focused update of the ESC Guidelines for the management of atrial fibrillation: An update of the 2010 ESC Guidelines for the management of atrial fibrillation. Developed with the special contribution of the European Heart Rhythm Association. Europace. 2012;14:1385-1413.

6. Chern-En C, Tsu-Juey W, Kwo-Chang U, Tze-Fan C, Kuan-Cheng C, Chun-Chieh W, et al. 2016 Guidelines of the Taiwan Heart Rhythm Society and the Taiwan Society of Cardiology for the management of atrial fibrillation. J Formos Med Assoc. 2016;115:893-952.

7. Epstein AE, Dimarco JP, Ellenbogen KA, Estes NAM, Freedman RA, Gettes LS, et al. 2012 ACCF/AHA/HRS focused update incorporated into the ACCF/AHA/HRS 2008 guidelines for device-based therapy of cardiac rhythm abnormalities: A report of the American College of Cardiology Foundation/American Heart Association Task Force on Practice Guidelines and the Heart Rhythm Society. Circulation. 2013;127:e283-e352.

8. Ferro JM, Bousser MG, Canhao P, Coutinho JM, Crassard I, Dentali F, et al. European Stroke Organization guideline for the diagnosis and treatment of cerebral venous thrombosis - endorsed by the European Academy of Neurology. Eur J Neurol. 2017;24:1203-1213.

9. Furie KL, Kasner SE, Adams RJ, Albers GW, Bush RL, Fagan SC, et al. Guidelines for the prevention of stroke in patients with stroke or transient ischemic attack: A guideline for healthcare professionals from the American Heart Association/American Stroke Association. Stroke. 2011;42:227-276.

10. Fuster V, Ryden LE, Cannom DS, Crijns HJ, Curtis AB, Ellenbogen KA, et al. 2011 ACCF/AHA/HRS Focused Updates Incorporated Into the ACC/AHA/ESC 2006 Guidelines for the Management of Patients With Atrial Fibrillation: A Report of the American College of Cardiology Foundation/American Heart Association Task Force on Practice Guidelines Developed in partnership with the European Society of Cardiology and in collaboration with the European Heart Rhythm Association and the Heart Rhythm Society. J Am Coll Cardiol. 2011;57:e101-e198.

11. Healey JS, Parkash R, Pollak T, Tsang T, Dorian P. Canadian cardiovascular society atrial fibrillation guidelines 2010: Etiology and initial investigations. Can J Cardiol. 2011;27:31-37.

12. January CT, Wann LS, Alpert JS, Calkins H, Cigarroa JE, Cleveland JC, et al. 2014 AHA/ACC/HRS guideline for the management of patients with atrial fibrillation: A report of the American College of Cardiology/American Heart Association Task Force on Practice Guidelines and the Heart Rhythm Society. Circulation. 2014;130:e199-e267.

13. January CT, Wann LS, Calkins H, Chen LY, Cigarroa JE, Cleveland JC, Jr., et al. 2019 AHA/ACC/HRS Focused Update of the 2014 AHA/ACC/HRS Guideline for the Management of Patients With Atrial Fibrillation. Circulation. 2019;140:e125-e151.

14. Jauch EC, Cucchiara B, Adeoye O, Meurer W, Brice J, Chan YY, et al. Part 11: adult stroke: 2010 American Heart Association Guidelines for Cardiopulmonary Resuscitation and Emergency Cardiovascular Care. Circulation. 2010;122:S818-828.

15. John Camm A, Lip GYH, De Caterina R, Savelieva I, Atar D, Hohnloser SH, et al. 2012 focused update of the ESC Guidelines for the management of atrial fibrillation. Eur Heart J. 2012;33:2719-2747.

16. Jung KH, Yu KH, Kim YD, Park JM, Hong KS, Rha JH, et al. Antithrombotic management of patients with nonvalvular atrial fibrillation and ischemic stroke or transient ischemic attack: Executive summary of the Korean Clinical Practice Guidelines for stroke. J Stroke. 2015;17:210-215.

17. *Kernan WN, Ovbiagele B, Black HR, Bravata DM, Chimowitz MI, Ezekowitz MD, et al. Guidelines for the prevention of stroke in patients with stroke and transient ischemic attack: A guideline for healthcare professionals from the American Heart Association/American Stroke Association. Stroke. 2014;45:2160-2236.

18. Kes VB, Cesarik M, Zavoreo I, Butkovic SS, Kes P, Basic-Jukic N, et al. Guidelines for diagnosis, therapy and follow up of Anderson-Fabry disease. [Croatian]. Acta Med Croatica. 2014;68:223-232.

19. Kuijpers T, Spencer FA, Siemieniuk RAC, Vandvik PO, Otto CM, Lytvyn L, et al. Patent foramen ovale closure, antiplatelet therapy or anticoagulation therapy alone for management of cryptogenic stroke? A clinical practice guideline. BMJ. 2018;362:k2515.

20. Lansberg MG, O'Donnell MJ, Khatri P, Lang ES, Nguyen-Huynh MN, Schwartz NE, et al. Antithrombotic and thrombolytic therapy for ischemic stroke: Antithrombotic therapy and prevention of thrombosis, 9th ed: American College of Chest Physicians evidence-based clinical practice guidelines. Chest. 2012;141:e601S-e636S.

21. Lip GYH, Banerjee A, Boriani G, Chiang Ce, Fargo R, Freedman B, et al. Antithrombotic Therapy for Atrial Fibrillation: CHEST Guideline and Expert Panel Report. Chest. 2018;154:1121-1201.

22. Liu X, Zhang S, Liu M, Wang Y, Wu J, Dong Q, et al. Chinese guidelines for endovascular management of ischemic cerebrovascular diseases. Interv Neurol. 2013;1:171-184.

23. Macle L, Cairns J, Leblanc K, Tsang T, Skanes A, Cox JL, et al. 2016 Focused Update of the Canadian Cardiovascular Society Guidelines for the Management of Atrial Fibrillation. Can J Cardiol. 2016;32:1170-1185.

24. Mitchell LB. Canadian Cardiovascular Society atrial fibrillation Guidelines 2010: Prevention and treatment of atrial fibrillation following cardiac surgery. Can J Cardiol. 2011;27:91-97.

25. Pontes-Neto OM, Cougo P, Martins SC, Abud DG, Nogueira RG, Miranda M, et al. Brazilian guidelines for endovascular treatment of patients with acute ischemic stroke. Arq Neuropsiquiatr. 2017;75:50-56.

26. Powers WJ, Derdeyn CP, Biller J, Coffey CS, Hoh BL, Jauch EC, et al. 2015 American Heart Association/American Stroke Association Focused Update of the 2013 Guidelines for the Early Management of Patients With Acute Ischemic Stroke Regarding Endovascular Treatment: A Guideline for Healthcare Professionals From the American Heart Association/American Stroke Association. Stroke. 2015;46:3020-3035.

27. Schellinger PD, Bryan RN, Caplan LR, Detre JA, Edelman RR, Jaigobin C, et al. Evidence-based guideline: The role of diffusion and perfusion MRI for the diagnosis of acute ischemic stroke: Report of the Therapeutics and Technology Assessment Subcommittee of the American Academy of Neurology. Neurology. 2010;75:177-185.

28. Skanes AC, Healey JS, Cairns JA, Dorian P, Gillis AM, McMurtry MS, et al. Focused 2012 Update of the Canadian Cardiovascular Society Atrial Fibrillation Guidelines: Recommendations for Stroke Prevention and Rate/Rhythm Control. Can J Cardiol. 2012;28:125-136.

29. Tracy CM, Epstein AE, Darbar D, Dimarco JP, Dunbar SB, Estes INAM, et al. 2012 ACCF/AHA/HRS focused update incorporated into the ACCF/AHA/HRS 2008 guidelines for device-based therapy of cardiac rhythm abnormalities: A report of the American College of Cardiology Foundation/American Heart Association Task Force on Practice Guidelines and the Heart Rhythm Society. J Am Coll Cardiol. 2013;61:e6-e75.

30. Wang Y, Liu M, Pu C. 2014 Chinese guidelines for secondary prevention of ischemic stroke and transient ischemic attack: Compiled by the Chinese Society of Neurology, Cerebrovascular Disease Group. Int J Stroke. 2017;12:302-320.

31. Wang YJ, Zhang SM, Zhang L, Wang CX. Chinese Guidelines for the Secondary Prevention of Ischemic Stroke and Transient Ischemic Attack 2010. CNS Neurosci Ther. 2012;18:93-101.

32. Wann LS, Curtis AB, Ellenbogen KA, Estes NAM, Ezekowitz MD, Jackman WM, et al. 2011 ACCF/AHA/HRS focused update on the management of patients with atrial fibrillation (Update on Dabigatran): A report of the American College of Cardiology

Foundation/American Heart Association Task Force on Practice Guidelines. Circulation. 2011;123:1144-1150.

33. Wann LS, Curtis AB, January CT, Ellenbogen KA, Lowe JE, Estes INAM, et al. 2011 ACCF/AHA/HRS focused update on the management of patients with atrial fibrillation (updating the 2006 guideline): A report of the American College of Cardiology Foundation/American Heart Association Task Force on Practice Guidelines. J Am Coll Cardiol. 2011;57:223-242.

34. Whitlock RP, Sun JC, Fremes SE, Rubens FD, Teoh KH. Antithrombotic and thrombolytic therapy for valvular disease: Antithrombotic therapy and prevention of thrombosis, 9th ed: American College of Chest Physicians evidence-based clinical practice guidelines. Chest. 2012;141:e576S-e600S.

35. You JJ, Singer DE, Howard PA, Lane DA, Eckman MH, Fang MC, et al. Antithrombotic therapy for atrial fibrillation: Antithrombotic Therapy and Prevention of Thrombosis, 9th ed: American College of Chest Physicians Evidence-Based Clinical Practice Guidelines. Chest. 2012;141:e531S-575S.

* although this guideline included a recommendation under a section on AF, the authors felt this was a more general secondary prevention strategy suggested for consideration rather than a prescriptive recommendation related to diagnostic investigations.

Summary of a published guideline (n=2)

1. Bryer A, Connor MD, Haug P, Cheyip B, Staub H, Tipping B, et al. The South African guideline for the management of ischemic stroke and transient ischemic attack: recommendations for a resource-constrained health care setting. Int J Stroke. 2011;6:349-354.

2. Venketasubramanian N, Pwee KH, Chen CP, Singapore Ministry of Health Clinical Practice Guidelines Workgroup on Stroke and Transient Ischaemic Attack. Singapore ministry of health clinical practice guidelines on stroke and transient ischemic attacks. Int J Stroke. 2011;6:251-258.

Newer version of the guideline available (n=5)

1. Camm AJ, Kirchhof P, Lip GYH, Schotten U, Savelieva I, Ernst S, et al. Guidelines for the management of atrial fibrillation. Eur Heart J. 2010;31:2369-2429.

2. Casaubon LK, Boulanger JM, Blacquiere D, Boucher S, Brown K, Goddard T, et al. Canadian Stroke Best Practice Recommendations: Hyperacute Stroke Care Guidelines, Update 2015. Int J Stroke. 2015;10:924-940.

3. Casaubon LK, Boulanger JM, Glasser E, Blacquiere D, Boucher S, Brown K, et al. Canadian Stroke Best Practice Recommendations: Acute Inpatient Stroke Care Guidelines, Update 2015. Int J Stroke. 2016;11:239-252.

4. Coutts SB, Wein TH, Lindsay MP, Buck B, Cote R, Ellis P, et al. Canadian Stroke Best Practice Recommendations: Secondary prevention of stroke guidelines, update 2014. Int J Stroke. 2015;10:282-291.

5. Jauch EC, Saver JL, Adams HP, Jr., Bruno A, Connors JJ, Demaerschalk BM, et al. Guidelines for the early management of patients with acute ischemic stroke: a guideline for healthcare professionals from the American Heart Association/American Stroke Association. Stroke. 2013;44:870-947.

Appendix E. Evidence assessment scales

Guideline	Grading system	Grade of Recommendation	Level of Evidence
National Institute for Health and Care Excellence (2019) Stroke and transient ischaemic attack in over 16s: diagnosis and initial management (NG128) [UK]	Grading of Recommendations Assessment Development and Evaluation (GRADE)	The wording used in the recommendations in the guideline (for example, words such as 'offer' and 'consider') denotes the certainty with which the recommendation is made (the strength of the recommendation).	**HIGH LEVEL:** Further research is very unlikely to change our confidence in the estimate of effect<br>**MODERATE LEVEL:** Further research is likely to have an important impact on our confidence in the estimate of effect and may change the estimate<br>**LOW LEVEL:** Further research is very likely to have an important impact on our confidence in the estimate of effect and is likely to change the estimate<br>**VERY LOW LEVEL:** Any estimate of effect is very uncertain
Powers et al. (2018) 2018 Guidelines for the Early Management of Patients With Acute Ischemic Stroke A Guideline for Healthcare Professionals From the American Heart Association/American Stroke Association	American College of Cardiology/American Heart Association Class of Recommendation and Level of Evidence	**CLASS I:** strong, benefit >>> risk<br>**CLASS IIa:** moderate, benefit >> risk<br>**CLASS IIb:** weak, benefit ≥ risk<br>**CLASS III:** no benefit (moderate), benefit = risk<br>**CLASS III:** harm (strong), risk > benefit	**LEVEL A**: High quality evidence from more then 1 RCT meta-analyses of high quality RCTs one or more RCTs corroborated by high quality registry studies<br>**LEVEL B-R (Randomized):** moderate quality evidence from 1 or more RCTs meta-analyses of moderate quality RCTs<br>**LEVEL B-NR (nonrandomized)**: moderate quality evidence from 1 or more well-designed, well-executed nonrandomized studies, observational studies or registry studies meta-analyses of such studies<br>**LEVEL C-LD (limited data)**: randomized or nonrandomized observational or registry studies with limitation of design or execution

			meta-analyses of such studies physiological or mechanistic studies in human subjects<br>**LEVEL C-EO (expert opinion):** consensus of expert opinion based on clinical experience
Boulanger et al. (2018) Canadian Stroke Best Practice Recommendations for Acute Stroke Management: Prehospital, Emergency Department, and Acute Inpatient Stroke Care, 6th Edition, Update 2018	Bespoke system	Uses levels of evidence only.	**LEVEL A:** Systematic reviews, meta-analyses, multiple homogenous randomized controlled trials<br>**LEVEL B:** Single randomized controlled trials, quasi-experimental design with large samples and power<br>**LEVEL C:** Weak evidence, expert opinion achieved by consensus
Wein et al. (2018) Canadian stroke best practice recommendations: Secondary prevention of stroke, sixth edition practice guidelines, update 2017	Bespoke system	Uses levels of evidence only.	**LEVEL A:** Evidence from a meta-analysis of randomized controlled trials or consistent findings from two or more randomized controlled trials. Desirable effects clearly outweigh undesirable effects or undesirable effects clearly outweigh desirable effects.<br>**LEVEL B:** Evidence from a single randomized controlled trial or consistent findings from two or more well-designed nonrandomized and/or noncontrolled trials, and large observational studies. Desirable effects outweigh or are closely balanced with undesirable effects or undesirable effects outweigh or are closely balanced with desirable effects.<br>**LEVEL C:** Writing group consensus and/or supported by limited research evidence.

			Desirable effects outweigh or are closely balanced with undesirable effects or undesirable effects outweigh or are closely balanced with desirable effects, as determined by writing group consensus.<br>**CLINICAL CONSIDERATION**: Reasonable practical advice provided by consensus of the writing group on specific clinical issues that are common and/or controversial and lack research evidence to guide practice
Joung et al. (2018) 2018 Korean Guideline of Atrial Fibrillation Management	Bespoke system/ no reference for system provided	**CLASS I:** Evidence and/or general agreement that a given treatment or procedure is beneficial, useful, effective.<br>**CLASS II:** Conflicting evidence and/or a divergence of opinion about the usefulness/efficacy of the given treatment or procedure.<br>**CLASS IIa:** Weight of evidence/opinion is in favour of usefulness/efficacy.<br>**CLASS IIb:** Usefulness/efficacy is less well established by evidence/opinion.<br>**CLASS III:** Evidence or general agreement that the given treatment or procedure is not useful/effective, and in some cases may be harmful.	**LEVEL A:** Data derived from multiple RCTs or meta-analyses.<br>**LEVEL B:** Data derived from a single RCT or large non-randomized studies.<br>**LEVEL C:** Consensus of opinion of the experts and/or small studies, retrospective studies, registries.
Stroke Foundation/ Australian Department of Health (2017) Clinical Guidelines for Stroke Management 2017	Grading of Recommendations Assessment Development and Evaluation (GRADE)	**STRONG RECOMMENDATIONS:** Where guideline authors are certain that the evidence supports a clear balance towards either desirable or undesirable effects;<br>**WEAK RECOMMENDATIONS:**	**HIGH LEVEL**: We are very confident that the true effect lies close to that of the estimate of the effect.<br>**MODERATE LEVEL:** We are moderately confident in the effect estimate: The true effect is likely to be close to the estimate of

		Where the guideline panel is uncertain about the balance between desirable and undesirable effects<br><br>These strong or weak recommendations can either be for or against an intervention**.**	the effect, but there is a possibility that it is substantially different<br>**LOW LEVEL:** Our confidence in the effect estimate is limited: The true effect may be substantially different from the estimate of the effect.<br>**VERY LOW LEVEL:** We have very little confidence in the effect estimate: The true effect is likely to be substantially different from the estimate of effect
Saric et al. (2016) Guidelines for the Use of Echocardiography in the Evaluation of a Cardiac Source of Embolism [American Society of Echocardiography]	No formal grading system used	Throughout these guidelines, recommendations are provided in the same format for all topics. There are three levels of recommendations: echocardiography recommended, echocardiography potentially useful, and echocardiography not recommended.	
Intercollegiate Stroke Working Party (2016) National Clinical Guideline for Stroke [Royal College of Physicians, UK]	No formal grading system used	For this guideline, as with previous editions, the Working Party has not adopted a hierarchical grading system for the 'strength' of recommendations. Instead, once all the recommendations were finalised, a formal consensus approach was used to identify the key recommendations in terms of their wider impact on stroke, and these are listed in the 'Key Recommendations' section.	
Kirchhof et al. (2016) 2016 ESC Guidelines for the management of atrial fibrillation developed in collaboration with EACTS	American College of Cardiology/American Heart Association Class of Recommendation and Level of Evidence	**CLASS I:** Evidence and/or general agreement that a given treatment or procedure is beneficial, useful, effective.<br>**CLASS II:** Conflicting evidence and/or a divergence of opinion about the usefulness/efficacy of the given treatment or procedure.	**LEVEL A:** Data derived from multiple randomized clinical trials or meta-analyses.<br>**LEVEL B:** Data derived from a single randomized clinical trial or large non-randomized studies.<br>**LEVEL C:** Consensus of opinion of the experts and/or small studies, retrospective studies, registries.

		**CLASS IIa:** Weight of evidence/opinion is in favour of usefulness/efficacy.<br>**CLASS IIb:** Usefulness/efficacy is less well established by evidence/opinion.<br>**CLASS III:** Evidence or general agreement that the given treatment or procedure is not useful/effective, and in some cases may be harmful.	
Ministry of Public Health (2016) Clinical Guidelines for the State of Qatar: The diagnosis and management of Stroke and transient ischemic attack [Qatar]	Bespoke system/ no reference for system provided	**GRADE A1 (RGA1):** Evidence demonstrates at least moderate certainty of at least moderate net benefit.<br>**GRADE A2 (RGA2):** Evidence demonstrates a net benefit, but of less than moderate certainty, and may consist of a consensus opinion of experts, case studies, and common standard care.<br>**GRADE B (RGB):** Evidence is insufficient, conflicting, or poor and demonstrates an incomplete assessment of net benefit vs harm; additional research is recommended.<br>**GRADE C1 (RGC1):**<br>vidence demonstrates a lack of net benefit; additional research is recommended.<br>**GRADE C2 (RGC2):**<br>Evidence demonstrates potential harm that outweighs benefit; additional research is recommended.<br>**RECOMMENDATION OF THE GDG (R-GDG):**	**LEVEL 1 (L1):** Meta-analyses; randomised controlled trials with meta-analysis; randomised controlled trials; systematic reviews.<br>**LEVEL 2 (L2):** Observational studies, examples include: cohort studies with statistical adjustment for potential confounders; cohort studies without adjustment; case series with historical or literature controls; uncontrolled case series; statements in published articles or textbooks.<br>**LEVEL 3 (L3):** Expert opinion; unpublished data, examples include: large database analyses; written protocols or outcomes reports from large practices.

		Recommended best practice on the basis of the clinical experience of the Guideline Development Group members.	
Verma et al. (2014) 2014 Focused Update of the Canadian Cardiovascular Society Guidelines for the Management of Atrial Fibrillation	Not described in the guideline		
National Institute for Health and Care Excellence (2014) Atrial fibrillation: management (CG180) [UK]	Grading of Recommendations Assessment Development and Evaluation (GRADE)	The wording used in the recommendations in the guideline (for example, words such as 'offer' and 'consider') denotes the certainty with which the recommendation is made (the strength of the recommendation).	**HIGH LEVEL:** Further research is very unlikely to change our confidence in the estimate of effect<br>**MODERATE LEVEL:** Further research is likely to have an important impact on our confidence in the estimate of effect and may change the estimate<br>**LOW LEVEL:** Further research is very likely to have an important impact on our confidence in the estimate of effect and is likely to change the estimate<br>**VERY LOW LEVEL:** Any estimate of effect is very uncertain
Oliveira-Filho et al. (2012) Guidelines for acute ischemic stroke treatment - part I [Brazil]	Oxford Classification	**GRADE A:** Systematic review (homogeneous) of RCT; or single RCT with narrow confidence interval; or therapeutic results of “all or nothing” type.<br>**GRADE B:** Systematic review (homogeneous) of cohort studies; or cohort study and RCT of lower quality; or	**LEVEL 1:** Randomized controlled clinical trial (RCT) or systematic review (SR) of RCT with clinical endpoints.<br>**LEVEL 2:** RCT or SR of lower quality: with substitute, validated endpoints; with subgroup analysis or with a posteriori hypotheses; with

		outcomes research or ecological study; or systematic review (homogeneous) of case-control studies; or case-control study.<br>**GRADE C:** Case reports (including cohort or case-control study of lower quality).<br>**GRADE D:** Expert opinion without critical evaluation, based on physiological or animal studies.	clinical endpoints, but with methodological flaws.<br>**LEVEL 3:** RCT with substitute, non-validated endpoints case-control studies.<br>**LEVEL 4:** Study with clinical endpoint, but with a higher potential bias (as in experiment without comparison group and other observational studies).<br>**LEVEL 5:** Representative forum or expert opinion without above mentioned evidence
Ministry of Health Malaysia, Academy of Medicine Malaysia, Malaysian Society of Neurosciences (2010) Management of Ischaemic Stroke (2nd Edition)	The level of recommendation and the grading of evidence used was adapted from the U.S/ Canadian Preventive Services Task Force, and the Guidelines for Clinical Practice Guideline, Ministry Of Health Malaysia 2003.	**GRADE A:** At least one meta analysis, systematic review, or randomized controlled trial (RCT), or evidence rated as good and directly applicable to the target population<br>**GRADE B:** Evidence from well conducted clinical trials, directly applicable to the target population, and demonstrating overall consistency of results; or evidence extrapolated from meta analysis, systematic review or RCT<br>**GRADE C:** Evidence from expert committee reports, or opinions and/or clinical experiences of respected authorities; indicates absence of directly applicable clinical studies of good quality"	**LEVEL I:** Evidence obtained from at least one properly randomized controlled trial<br>**LEVEL II – 1:** Evidence obtained from well-designed controlled trials without randomization<br>**LEVEL II – 2:** Evidence obtained from well-designed cohort or case-control analytic studies, preferably from more than one centre or research group<br>**LEVEL II – 3:** Evidence obtained from multiple time series with or without the intervention. Dramatic results in uncontrolled experiments (such as the results of the introduction of penicillin treatment in the 1940s) could also be regarded as this type of evidence<br>**LEVEL III:** Opinions of respected authorities, based on clinical experience, descriptive studies and case reports; or reports of expert committees"

Bryer et al. (2010) South African guideline for management of ischaemic stroke and transient ischaemic attack 2010: a guideline from the South African Stroke Society (SASS) and the SASS Writing Committee.	European Stroke Organisation system	**Evidence classification scheme for a diagnostic measure**<br><br>**CLASS I:** A prospective study in a broad spectrum of persons with the suspected condition, using a 'gold standard' for case definition, where the test is applied in a blinded evaluation, and enabling the assessment of appropriate tests of diagnostic accuracy.<br>**CLASS II:** A prospective study of a narrow spectrum of persons with the suspected condition, or a well-designed retrospective study of a broad spectrum of persons with an established condition (by 'gold standard') compared with a broad spectrum of controls, where test is applied in a blinded evaluation, and enabling the assessment of appropriate tests of diagnostic accuracy.<br>**CLASS III:** Evidence provided by a retrospective study where either persons with the established condition or controls are of a narrow spectrum, and where test is applied in a blinded evaluation.<br>**CLASS IV:** Evidence from uncontrolled studies, case series, case reports, or expert opinion.	**LEVEL A:** Established as useful/predictive or not useful/ predictive for a diagnostic measure or established as effective, ineffective or harmful for a therapeutic intervention; requires at least one convincing class I study or at least two consistent, convincing class II studies.<br>**LEVEL B:** Established as useful/predictive or not useful/predictive for a diagnostic measure or established as effective, ineffective or harmful for a therapeutic intervention; requires at least one convincing class II study or overwhelming class III evidence.<br>**LEVEL C:** Established as useful/predictive or not useful/predictive for a diagnostic measure or established as effective, ineffective or harmful for a therapeutic intervention; requires at least two class III studies.<br>**GOOD CLINICAL PRACTICE (GCP):** Recommended best practice based on the experience of the guideline development group. Usually based on class IV evidence indicating large clinical uncertainty; such GCP points can be useful for health workers.
Ministry of Health (2009) Stroke and Transient Ischaemic Attacks. Assessment,	Bespoke system/ no reference for system provided	**GRADE A:** At least one meta-analysis, systematic review of RCTs, or RCT rated as 1+ + and directly applicable to the target population; or A body of evidence	**LEVEL 1++:** High quality meta-analyses, systematic reviews of randomised controlled trials (RCTs), or RCTs with a very low risk of bias.

Investigation, Immediate Management and Secondary Prevention. [Singapore]		consisting principally of studies rated as 1+, directly applicable to the target population, and demonstrating overall consistency of results<br>**GRADE B:** A body of evidence including studies rated as 2++, directly applicable to the target population, and demonstrating overall consistency of results; or Extrapolated evidence from studies rated as 1+ + or 1+<br>**GRADE C:** A body of evidence including studies rated as 2+, directly applicable to the target population and demonstrating overall consistency of results; or Extrapolated evidence from studies rated as 2+ +<br>**GRADE D**: Evidence level 3 or 4; or Extrapolated evidence from studies rated as 2+<br>**GOOD PRACTICE POINT (GPP):** Recommended best practice based on the clinical experience of the guideline development group.	**LEVEL 1+:** Well conducted meta-analyses, systematic reviews of RCTs, or RCTs with a low risk of bias.<br>**LEVEL 1-:** Meta-analyses, systematic reviews of RCTs, or RCTs with a high risk of bias.<br>**LEVEL 2++:** High quality systematic reviews of case control or cohort studies. High quality case control or cohort studies with a very low risk of confounding or bias and a high probability that the relationship is causal<br>**LEVEL 2+:** Well conducted case control or cohort studies with a low risk of confounding or bias and a moderate probability that the relationship is causal<br>**LEVEL 2-:** Case control or cohort studies with a high risk of confounding or bias and a significant risk that the relationship is not causal<br>**LEVEL 3:** Non-analytic studies, e.g. case reports, case series<br>**LEVEL 4:** Expert opinion
**Consensus documents/statements**			
Gorenek et al. (2017) Device-detected subclinical atrial tachyarrhythmias: definition, implications and management—an European Heart Rhythm Association	European Heart Rhythm Association system	A ‘green heart’ indicates a recommended statement or recommended/indicated treatment or procedure and is based on at least one randomized trial, or is supported by strong observational evidence that it is beneficial and effective.	No separate definitions of Level of Evidence

(EHRA) consensus document, endorsed by Heart RhythmSociety (HRS), Asia Pacific Heart Rhythm Society (APHRS) and Sociedad Latinoamericana de Estimulacióín Cardíaca y Electrofisiología (SOLEACE).		A ‘yellow heart’ indicates that general agreement and/or scientific evidence favouring a statement or the usefulness/efficacy of a treatment or procedure may be supported by randomized trials based on small number of patients or not widely applicable.<br><br>Treatment strategies for which there has been scientific evidence that they are potentially harmful and should not be used are indicated by a ‘red heart’	
Prasad et al. (2014) Recommendations for the Early Management of Acute Ischemic Stroke: A Consensus Statement for Healthcare Professionals from the Indian Stroke Association	None reported	None reported	None reported
Wintermark et al. (2013) Imaging Recommendations for Acute Stroke and Transient Ischemic Attack Patients: A Joint Statement by the American Society of Neuroradiology, the	Oxford Centre for Evidence-based Medicine Levels of Evidence.	Used levels of evidence only.	**Levels of evidence specific to the accuracy of diagnostic tests.**<br><br>**LEVEL Ia:** Systematic review (with homogeneity)[b] of Level 1 studies. Level 1 studies are studies: that use a blind comparison of the test with a validated reference standard; in a sample of patients that

American College of Radiology and the Society of NeuroInterventional Surgery			reflects the population to whom the test would apply<br>**LEVEL Ib:** Level 1 studies<br>**LEVEL II:** Level 2 studies; systematic reviews of Level 2 studies. Level 2 studies are studies that have only 1 of the following: narrow population (the sample does not reflect the population to whom the test would apply); use a poor reference standard (defined as that where the "test" is included in the "reference," or where the "testing" affects the "reference"); the comparison between the test and reference standard is not blind; case– control studies<br>**LEVEL III:** Level 3 studies; systematic reviews of Level 3 studies. Level 3 studies are studies that have at least 2 or 3 of the features listed above.<br>**LEVEL IV:** Consensus, expert committee reports or opinions, and/or clinical experience without explicit critical appraisal; or based on physiology, bench research, or "first principles"
Pepi et al. (2010) Recommendations for echocardiography use in the diagnosis and management of cardiac sources of embolism [European Association of Echocardiography (EAE)]	No formal grading system used	Because of the diverse nature of the topics and the absence of objective rating levels of evidence (mainly due to gaps in current knowledge in several fields), it was not possible to provide a systematic uniform summary of recommendations in all chapters. On the basis of all these considerations, the writing group decided to avoid levels of recommendations and maintain only the term 'Recommendation'. This implies an appropriate method recommended for all patients with a suspected of cardiac source of embolism. (p.462)	

Irimia et al. (2011) Use of imaging in cerebrovascular disease [European Federation of Neurological Societies]	Not described in the document		
Summers et al. (2009) Comprehensive Overview of Nursing and Interdisciplinary Care of the Acute Ischemic Stroke Patient: A Scientific Statement From the American Heart Association	American College of Cardiology/American Heart Association Class of Recommendation and Level of Evidence	**CLASS I:** Benefit >>> risk.<br>Treatment/ procedure SHOULD be performed/ administrated<br>**CLASS IIa:** Benefit>> risk.<br>It is REASONABLE to perform/ admin treatment<br>**CLASS IIb:** Benefit >= risk.<br>Procedure/ treatment MAY BE considered<br>**CLASS III:** Risk >= benefit.<br>Procedure/ treatment SHOULD NOT be performed AS IT IS NOT HELPFULAND MY BE HARMFUL	**LEVEL A:** Multiple populations evaluated; data derived from multiple randomized clinical trials or meta-analyses<br>**LEVEL B:** Limited populations evaluated; data derived from a single randomized trial or nonrandomized studies<br>**LEVEL C:** Very limited populations evaluated; only consensus opinion of experts, case studies or standard of care
Latchaw et al. (2009) Recommendations for Imaging of Acute Ischemic Stroke: A Scientific Statement From the American Heart Association	American College of Cardiology/American Heart Association Class of Recommendation and Level of Evidence	**CLASS I:** Conditions for which there is evidence for and/or general agreement that a procedure or treatment is beneficial, useful, and effective<br>**CLASS II:** Conditions for which there is conflicting evidence and/or a divergence of opinion about the usefulness/efficacy of a procedure or treatment<br>**CLASS IIa:** Weight of evidence/opinion is in favor of usefulness/efficacy<br>**CLASS IIb:** Usefulness/efficacy is less well established by evidence/opinion<br>**Class III:** Conditions for which there is evidence and/or general agreement that a	**LEVEL A:** Data derived from multiple randomized clinical trials or meta-analyses<br>**LEVEL B:** Data derived from a single randomized trial or nonrandomized studies<br>**LEVEL C:** Only consensus opinion of experts, case studies, or standard-of-care

		procedure/treatment is not useful/effective and in some cases may be harmful	

Table I. Commentary in the stroke management guidelines/statements on establishing stroke etiology

Guideline	Relevant text
National Institute for Health and Care Excellence (2019) Stroke and transient ischaemic attack in over 16s: diagnosis and initial management (NG128) [UK]	No explicit statement about establishing stroke etiology.<br>Two relevant recommendations related to brain imaging in diagnostic workup (see Table II).
Powers et al. (2018) 2018 Guidelines for the Early Management of Patients With Acute Ischemic Stroke A Guideline for Healthcare Professionals From the American Heart Association/American Stroke Association	No explicit statement about establishing stroke etiology.
Boulanger et al. (2018) Canadian Stroke Best Practice Recommendations for Acute Stroke Management: Prehospital, Emergency Department, and Acute Inpatient Stroke Care, 6th Edition, Update 2018	Explicit statement that identifying nature and mechanism of stroke is a principal aim of acute stroke care:<br>This section includes recommendations related to management of acute stroke patients once diagnosis is confirmed and decisions made regarding early acute stroke treatments. It involves all direct care, investigations, interventions, service delivery, and interactions occurring during the time a person who has had a stroke is admitted to inpatient care within an acute care hospital. The principal aims of this phase of care are **to identify the nature and mechanism of stroke**, prevent stroke complications, promote early recovery, and (in the case of severest strokes) provide palliation or end-of-life care. This acute phase of care is usually considered to have ended either at the time of acute stroke unit discharge or by 30 days of hospital admission. (p.971)
Stroke Foundation/Australian Department of Health (2017) Clinical Guidelines for Stroke Management 2017	Explicit statement that the role of investigations is to determine the cause of the stroke. A list of routine investigations provided, and it is suggested that further

	investigations may be necessary in instances where these do not establish cause. No further details provided on specific pathways of investigation.<br><br>Once a clinical diagnosis of stroke has been made, additional investigations are used to confirm the diagnosis and **to determine the cause of the event**, specifically if it is cardiac or carotid in origin. Routine investigations should include full blood count, electrolytes, erythrocyte sedimentation rate, C-reactive protein, renal function, cholesterol and glucose levels, although direct evidence is lacking for each of these investigations. An ECG should also be conducted routinely to detect AF. If clinical history, imaging and routine investigations do not adequately identify the underlying cause then further investigations may be warranted. (p.32)
Intercollegiate Stroke Working Party (2016) National Clinical Guideline for Stroke [Royal College of Physicians, UK]	Explicit statement about the need to conduct further investigation of stroke mechanism if not established through initial investigation.<br><br>In about a quarter of people with stroke, and more commonly in younger age groups, no cause is evident on initial investigation. Other causes that should be considered include paroxysmal atrial fibrillation (PAF), intracranial arterial disease, cervical artery dissection, antiphospholipid syndrome and other prothrombotic conditions, and patent foramen ovale (PFO). In younger people in whom no cause is identified with a history of venous or arterial thrombosis or early miscarriage, a thrombophilia screen should be performed. (p.99)
Ministry of Public Health (2016) Stroke and transient ischemic attack [Qatar]	No explicit statement about establishing stroke etiology.
Oliveira-Filho et al. (2012) Guidelines for acute ischemic stroke treatment - part I [Brazil]	No explicit statement about establishing stroke etiology.

Ministry of Health Malaysia, Academy of Medicine Malaysia, Malaysian Society of Neurosciences (2010) Management of Ischaemic Stroke (2nd Edition)	Explicit statements about the need to identify underlying cause of the stroke with a view to guiding secondary prevention.<br><br>Classification of stroke has numerous implications during immediate stroke supportive care and rehabilitation, for prognostic purposes, guides cost effective investigations for underlying cause as well as aids decisions for therapy and secondary stroke prevention strategies. Furthermore, classifications are useful in setting up stroke registries and data banks as well as for epidemiological studies. (p.1)<br><br>The diagnosis should provide answers to the following questions:<br>1. What is the neurological deficit?<br>2. Where is the lesion?<br>3. What is the lesion?<br>4. Why has the lesion occurred?<br>5. What are the potential complications and prognosis? (p.2)<br><br>The guideline provides a stroke pathophysiology algorithm which details the potential underlying causes of a stroke which merit investigation and provides two extensive lists detailing investigations that are mandatory and those which should be completed in selection patients, one for general stroke cases and one for cases of stroke in young adults. There were no relevant formal recommendations identified in this guideline as the focus of such recommendations tended to be on treatment, with the content on establishing stroke etiology presented in the main text and appendices.

Bryer et al. (2010) South African guideline for management of ischaemic stroke and transient ischaemic attack 2010: A guideline from the South African Stroke Society (SASS) and the SASS Writing Committee	Explicit statements about the need to identify underlying cause of the stroke during evaluation with a view to guiding secondary prevention.<br><br>Initial evaluation of a suspected stroke patient entails checking vital signs and stabilisation of the patient, followed by assessment of neurological deficit and co-morbidities. Goals of this assessment include:<br><br>• determining whether patient has had a stroke<br>• identifying whether or not the patient is a suitable candidate for emergency interventional therapy with agents such as tPA<br>• excluding stroke mimics (i.e. other conditions with stroke-like symptoms)<br>• identifying other conditions that require immediate intervention (e.g. hypoglycaemia – urgent blood glucose assessment and treat if hypoglycaemic)<br>• determining potential causes of the stroke for early secondary prevention. (p.762)<br><br>A list of investigations is provided in the guideline, divided into those which should be performed for all patients, and those which may be required on selected patients. (p.762)

Ministry of Health (2009) Stroke and transient ischaemic attacks. Assessment, investigation, immediate management and secondary prevention. [Singapore]	Explicit statements about the need to identify underlying cause of the stroke during evaluation with a view to guiding secondary prevention.<br><br>The results of assessment and investigation should answer the following questions:<br><br>(1) Is this a vascular event, i.e. a stroke or transient ischaemic attack (TIA)?<br>(2) Which part of the brain is affected?<br>(3) Is it an ischaemic or haemorrhagic vascular event?<br>(4) What is the cause of the vascular event?<br>(5) What functional and social problems does this cause the patient?<br>(6) What other medical problems co-exist with and affect the management of the stroke?<br>(7) What facilities are required for the management of this patient? (p.11)<br><br>Investigations are undertaken:<br><br>• to confirm the nature of the vascular event [question (1) above] and to elucidate upon the underlying cause [questions (3) and (4)]<br>• to determine the appropriate strategy for acute intervention and secondary prevention<br>• to identify prognostic factors. (p.12)

Consensus documents/statements	Relevant text
Prasad et al. (2014) Recommendations for the Early Management of Acute Ischemic Stroke: A Consensus Statement for Healthcare Professionals from the Indian Stroke Association [India]	Statements about the need to identify underlying cause of the stroke during evaluation with a view to guiding secondary prevention.<br><br>Patient history should be comprehensive and should be taken within 5 minutes. The overall aim of collecting patient history is not only to identify a possible stroke but also to exclude stroke mimics (conditions with stroke-like symptoms, e.g., primary tumor of brain, metastatic neoplasm of brain, meningoencephalitis, thyrotoxicosis, hypoglycemia [Table 8]), and identify the need for immediate interventions and determine potential causes of stroke for secondary prevention measures.<br><br>A thorough general physical examination needs to be performed to identify other potential causes of patients' symptoms and an ischemic stroke, coexisting comorbidities, or issues that may affect the management of an ischemic stroke. (no page number)<br><br>A list of investigations is provided differentiating those to be performed for all patients, and those which may be required for selected patients.
Summers et al. (2009) Comprehensive Overview of Nursing and Interdisciplinary Care of the Acute Ischemic Stroke Patient: A Scientific Statement From the American Heart Association	Explicit statements about the need to identify underlying cause of the stroke during evaluation with a view to guiding secondary prevention.<br><br>During the acute care phase, nursing care should focus on continued stabilization of the stroke patient through frequent evaluation of neurological status, blood pressure management, and prevention of complications. Medical management focuses on establishing the cause or etiology of AIS, prevention of treatment-related complications, and evaluation of secondary prevention strategies. There is considerable evidence that dedicated stroke teams, units, and coordinated care improve clinical outcomes in the acute care phase.[5,7,57,114–121] (p.2921)

Table II. Recommendations for brain imaging

Guideline	Recommendations
National Institute for Health and Care Excellence (2019) Stroke and transient ischaemic attack in over 16s: diagnosis and initial management (NG128) [UK]	Perform brain imaging immediately with a non-enhanced CT for people with suspected acute stroke if any of the following apply:<br>• indications for thrombolysis or thrombectomy<br>• on anticoagulant treatment<br>• a known bleeding tendency<br>• a depressed level of consciousness (Glasgow Coma Score below 13)<br>• unexplained progressive or fluctuating symptoms<br>• papilloedema, neck stiffness or fever<br>• severe headache at onset of stroke symptoms<br>If thrombectomy might be indicated, perform imaging with CT contrast angiography following initial non-enhanced CT. Add CT perfusion imaging (or MR equivalent) if thrombectomy might be indicated beyond 6 hours of symptom onset. **[reflected in wording]**<br>Perform scanning as soon as possible and within 24 hours of symptom onset in everyone with suspected acute stroke without indications for immediate brain imaging. **[reflected in wording]**
Powers et al. (2018) 2018 Guidelines for the Early Management of Patients With Acute Ischemic Stroke A Guideline for Healthcare Professionals From the American Heart Association/American Stroke Association	All patients admitted to hospital with suspected acute stroke should receive brain imaging evaluation on arrival to hospital. In most cases, noncontrast CT (NCCT) will provide the necessary information to make decisions about acute management. **[Class I, Level B-NR]**
Boulanger et al. (2018) Canadian Stroke Best Practice Recommendations for Acute Stroke Management: Prehospital, Emergency	VERY HIGH risk for recurrent stroke (symptom onset within last 48 h): Urgent brain imaging (computed tomography (CT) or magnetic resonance imaging (MRI)) and non-

Department, and Acute Inpatient Stroke Care, 6th Edition, Update 2018	invasive vascular imaging (CT angiography (CTA) or MR angiography (MRA) from aortic arch to vertex) should be completed as soon as possible within 24 h **[Level B]**<br><br>All patients with suspected acute ischemic stroke who arrive within 4.5 h and are potentially eligible for intravenous thrombolysis (refer to criteria in Box 4A) should undergo immediate brain imaging with non-contrast CT (NCCT) without delay to determine eligibility for thrombolysis. **[Level A]**<br><br>Brain imaging (CT or MRI) and non-invasive vascular imaging (CTA or MRA from aortic arch to vertex) should be completed as appropriate and within time frames based on triage category and severity*. **[Level B]**<br><br>MRI is superior to CT scan in terms of diagnostic sensitivity for small strokes and may provide additional information that could guide diagnosis, prognosis, and management decision-making. Decisions regarding MRI scanning should be based on MRI access, availability, and timing of appointments. **[Not provided]**
Wein et al. (2018) Canadian stroke best practice recommendations: Secondary prevention of stroke, sixth edition practice guidelines, update 2017	Brain imaging (CT or MRI) and non-invasive vascular imaging (CTA or MRA from aortic arch to vertex) should be completed within time frames based on triage category. **[Level B]**
Stroke Foundation/Australian Department of Health (2017) Clinical Guidelines for Stroke Management 2017	In patients with suspected stroke and TIA, MRI is more sensitive and specific than non-contrast CT and is the preferred modality when diagnostic confirmation is required. **[Weak recommendation, High quality of evidence]**<br><br>If using CT to identify hyperdense thrombus, thin slice (< 2 mm) non-contrast CT should be used rather than the standard 5 mm slices to improve diagnostic sensitivity for vessel occlusion. **[Strong recommendation, High quality of evidence]**<br><br>CT perfusion imaging may be used in addition to routine imaging to improve diagnostic and prognostic accuracy. **[Weak recommendation, High quality of evidence]**

Intercollegiate Stroke Working Party (2016) National Clinical Guideline for Stroke [Royal College of Physicians, UK]	Patients with suspected acute stroke should receive brain imaging urgently and at most within 1 hour of arrival at hospital. **[Reflected in wording]**<br>MRI with stroke-specific sequences (diffusion-weighted imaging, T2*) should be performed in patients with suspected acute stroke when there is diagnostic uncertainty. **[Reflected in wording]**
Oliveira-Filho et al. (2012) Guidelines for acute ischemic stroke treatment - part I [Brazil]	For patients with acute stroke, an urgent noncontrast head CT is recommended **[Grade A; Level 1A]** or, alternatively, cranial MRI with the inclusion of diffusion and gradient echo sequences **[Grade A, Level IB].**
Bryer et al. (2010) South African guideline for management of ischaemic stroke and transient ischaemic attack 2010: A guideline from the South African Stroke Society (SASS) and the SASS Writing Committee	In patients with suspected stroke or TIA, urgent cranial CT is recommended **[Class I]** or, MRI **[Class II, Level A].**<br>If MRI is used, the inclusion of diffusion-weighted imaging (DWI) and T2-weighted gradient echo sequences is recommended. **[Class II, Level A]**
Ministry of Health (2009) Stroke and transient ischaemic attacks. Assessment, investigation, immediate management and secondary prevention. [Singapore]	All patients with transient ischaemic attack or an acute stroke syndrome should have a computed tomography or magnetic resonance imaging brain scan as soon as possible **[Level 2+, Grade C]**, preferably within 24 hours **[GPP]**.
**Consensus documents/statements**	**Recommendations**
Prasad et al. (2014) Recommendations for the Early Management of Acute Ischemic Stroke: A Consensus Statement for Healthcare Professionals from the Indian Stroke Association [India]	Brain imaging should be performed immediately for patients with suspected stroke. A non-contrast CT scan is recommended as the initial imaging should be sufficient in most cases. In centers with multislice CT scanners, CT angiography along with a CT scan should be performed. **[not provided]**
Wintermark et al. (2013) Imaging recommendations for acute stroke and transient	When revascularization therapy is not indicated or available, multimodal neuroimaging of the brain and cerebrovasculature with MR imaging should be performed to confirm the

ischemic attack patients: a joint statement by the American Society of Neuroradiology, the American College of Radiology and the Society of NeuroInterventional Surgery [American Society of Neuroradiology]	diagnosis of stroke, identify the underlying etiology, and assess immediate complications and risk of future stroke. **[not provided]**<br><br>Multimodal CT, including NCCT and CTA and possibly PCT, should be reserved for patients who have contraindications to MR imaging, or if MR imaging is not available. **[not provided]**
Irimia et al. (2011) Use of imaging in cerebrovascular disease [European Federation of Neurological Societies]	Conventional CT of the head is the examination most frequently used for the emergent evaluation of patients with acute stroke because of its wide availability and usefulness. **[Class II, Level B]**<br><br>In conjunction with MRI and magnetic resonance angiography (MRA), perfusion and diffusion MR are very helpful for the evaluation of patients with acute ischaemic stroke**.** **[Class I, Level A]**<br><br>Magnetic resonance imaging has a higher sensitivity than conventional CT and results in lower inter-rater variability in the diagnosis of ischaemic stroke within the first hours of stroke onset. **[Class I, Level A]**<br><br>MRI has a higher sensitivity than conventional CT for the documentation of infarction within the first hours of stroke onset, lesions in the posterior fossa, identification of small lesions, and documentation of vessel occlusion and brain oedema. **[Class I, Level A]**
Summers et al. (2009) Comprehensive Overview of Nursing and Interdisciplinary Care of the Acute Ischemic Stroke Patient: A Scientific Statement From the American Heart Association	CT or MRI of the head should be performed emergently in patients who present to the ED within the 3-h window. [**Class I, Level A]**

*Triage categories:

- VERY HIGH risk for recurrent stroke (symptom onset within last 48 h): investigations completed as soon as possible within 24hours.
- HIGH risk for recurrent stroke (symptom onset between 48 h and two weeks): investigations ideally initiated within 24h of first contact.
- MODERATE (INCREASED) risk for recurrent stroke (symptom onset between 48 h and two weeks): investigations within two weeks of first contact.
- LOWER risk for recurrent stroke (time lapse since symptom onset greater than two weeks): investigations ideally within one month of symptom onset.

Table III. Recommendations for vascular imaging

Guideline	Recommendations
Powers et al. (2018) 2018 Guidelines for the Early Management of Patients With Acute Ischemic Stroke A Guideline for Healthcare Professionals From the American Heart Association/American Stroke Association	For patients who otherwise meet criteria for EVT, a noninvasive intracranial vascular study is recommended during the initial imaging evaluation of the acute stroke patient, but should not delay IV alteplase if indicated. For patients who qualify for IV alteplase according to guidelines from professional medical societies, initiating IV alteplase before noninvasive vascular imaging is recommended for patients who have not had noninvasive vascular imaging as part of their initial imaging assessment for stroke. Noninvasive intracranial vascular imaging should then be obtained as quickly as possible. **[Class I, Level A]**
Boulanger et al. (2018) Canadian Stroke Best Practice Recommendations for Acute Stroke Management: Prehospital, Emergency Department, and Acute Inpatient Stroke Care, 6th Edition, Update 2018	Brain imaging (CT or MRI) and non-invasive vascular imaging (CTA or MRA from aortic arch to vertex) should be completed as appropriate and within time frames based on triage category and severity*. **[Level B]**<br><br>CTA including extracranial and intracranial vasculature from aortic arch to vertex, which can be performed at the time of initial brain CT, is recommended as an ideal way to assess both the extracranial and intracranial circulation. **[Level B]**<br><br>Vascular imaging is recommended to identify significant symptomatic extracranial carotid artery stenosis for which patients should be referred for possible carotid revascularization. **[Level A]**<br><br>Carotid ultrasound (for extracranial vascular imaging) and MR angiography are acceptable alternatives to CTA, and selection should be based on immediate availability, and patient characteristics. **[Level C]**<br><br>VERY HIGH risk for recurrent stroke (symptom onset within last 48 h): Urgent brain imaging (computed tomography (CT) or magnetic resonance imaging (MRI)) and non-invasive vascular imaging (CT angiography (CTA) or MR angiography (MRA) from aortic arch to vertex) should be completed as soon as possible within 24 h. **[Level B]**

	All patients with suspected acute ischemic stroke who arrive within 6 h and are potentially eligible for EVT (refer to criteria in Box 4B and Section 5) should undergo immediate brain imaging non-contrast CT and CT angiography (CTA) without delay, from arch-to-vertex including the extra- and intra-cranial circulation, to identify large vessel occlusions eligible for endovascular thrombectomy. **[Level A]**
Wein et al. (2018) Canadian stroke best practice recommendations: Secondary prevention of stroke, sixth edition practice guidelines, update 2017	CT angiography including extracranial and intracranial vasculature from aortic arch to vertex, which can be performed at the time of initial brain CT, is recommended as an ideal way to assess both the extracranial and intracranial circulation. **[Level B]**<br><br>Vascular imaging is recommended to identify significant symptomatic extracranial carotid artery stenosis for which patients should be referred for possible carotid revascularization. **[Level A]**
Stroke Foundation/ Australian Department of Health (2017) Clinical Guidelines for Stroke Management 2017	In ischaemic stroke and TIA patients, routinely imaging the entire vasculature from aortic arch to cerebral vertex with CTA or MRA is encouraged to improve diagnosis, recognition of stroke aetiology and assessment of prognosis. **[Practice point]**<br><br>All patients who would potentially be candidates for endovascular thrombectomy should have vascular imaging from aortic arch to cerebral vertex (CTA or MRA) to establish the presence of vascular occlusion as a target for thrombectomy and to assess proximal vascular access. **[Strong recommendation, low quality of evidence]**<br><br>All other patients with carotid territory symptoms who would potentially be candidates for carotid re-vascularisation should have early vascular imaging to identify stenosis in the ipsilateral carotid artery. CT angiography (if not already performed as part of assessment for reperfusion therapies), Doppler ultrasound or contrast-enhanced MR angiography are all reasonable options depending on local experience and availability. **[Strong recommendation, low quality of evidence]**
Intercollegiate Stroke Working Party (2016) National Clinical Guideline for Stroke [Royal College of Physicians, UK]	Any patient suspected of cervical artery dissection should be investigated with CT or MR including angiography. **[Reflected in wording]**

Ministry of Public Health (2016) Stroke and transient ischemic attack [Qatar]	Carotid artery imaging:<br>• All people with suspected anterior circulation stroke or TIA, who after specialist assessment are considered as candidates for carotid endarterectomy.<br>• Carotid duplex ultrasound should be performed within 24-48 hours.<br>• High risk patients (ABCD^2 of ≥3) should have carotid imaging in <24 hours.<br>• Carotid endarterectomy should be considered where carotid stenosis is ≥70-99%.<br>• In selected patients, carotid endarterectomy can also be performed in patients with stenosis of 60-70%.<br>• Other revascularisation procedures can be considered in younger patients.<br>**[Recommendation of the Guideline Development Group]**
Bryer et al. (2010) South African guideline for management of ischaemic stroke and transient ischaemic attack 2010: A guideline from the South African Stroke Society (SASS) and the SASS Writing Committee	In patients with TIA, minor stroke or early spontaneous recovery, immediate diagnostic work-up, including urgent vascular imaging (carotid ultrasound, CT angiography, or MR angiography) is recommended. **[Class I, Level A]**
**Consensus documents/statements**	**Recommendations**
Wintermark et al. (2013) Imaging recommendations for acute stroke and transient ischemic attack patients: a joint statement by the American Society of Neuroradiology, the American College of Radiology and the Society of NeuroInterventional Surgery [American Society of Neuroradiology]	For patients who are outside the time window for acute reperfusion therapies (4.5 hours at sites where only IV tPA is being considered; 8 hours at sites where endovascular therapy is considered) and for patients with TIAs, emphasis is on secondary prevention and their imaging work-up should be focused on vascular imaging (CTA, MRA or Doppler-ultrasound [DUS]) to assess carotid arteries as a possible cause of the ischemic stroke, with secondary prevention in mind. If MRA is obtained, it makes sense to concurrently obtain MR imaging with DWI, FLAIR, and GRE/SWI. Echocardiography should also be obtained to assess for cardiac sources.<br><br>In acute stroke patients, vascular imaging should be performed to evaluate the mechanism of stroke and assess risk of future stroke. Overall, vascular imaging with DUS, CTA, MRA, or DSA has good agreement.
Irimia et al. (2011) Use of imaging in cerebrovascular disease [European Federation of Neurological Societies]	Non-invasive imaging methods are increasingly accepted as replacements of digital substraction angiography (DSA) in the evaluation of carotid stenosis prior to endarterectomy. **[Class IV, GCPP]**

	TCD is the only imaging technique that allows detection of circulating emboli, even in asymptomatic patients. **[Class II, Level A]**<br><br>Although MRA has slightly higher sensitivity and specificity than ultrasonography (US) to determine carotid stenosis and occlusion, the usefulness of either procedure may be determined by other factors, such as availability. **[Class II, Level B]**<br><br>Computed tomography angiography (CTA) has a sensitivity and specificity similar to MR for carotid occlusion and similar to US for the detection of severe stenosis. **[Class II, Level B]**<br><br>TCD can detect cerebral emboli and impaired cerebral haemodynamics. The presence of embolic signals with carotid stenosis predicts early recurrent stroke risk **[Class II, Level A].** The detection of impaired cerebral haemodynamics in carotid occlusion may identify a group at high risk of recurrent stroke **[Class III, Level B].**
Latchaw et al. (2009) Recommendations for Imaging of Acute Ischemic Stroke: A Scientific Statement From the American Heart Association	A vascular study is probably indicated during the initial imaging evaluation of the acute stroke patient, even if within 3 hours from ictus, to further determine the diagnosis of acute stroke, if such a study does not unduly delay the administration of intravenous tPA and if an endovascular team is available. **[Class IIa, Level B]**<br><br>For the detection of vascular stenoses and aneurysms, CTA and DSA are recommended **[Class I, Level A]** whereas MRA is less accurate but can be useful. **[Class IIa, Level A]**<br><br>CTA-SI exceeds NECT and may approach DWI for the detection of large ischemic regions, and although it is less effective for demonstrating small lesions or those in the posterior fossa, it is reasonable to use. **[Class IIa, Level B]**<br><br>Acute large-vessel intracranial thrombus is very accurately detected by CTA, DSA, and MRA. Each of these modalities far surpasses the sensitivity of nonvascular studies such as NECT, FLAIR, or gradient-echo MRI, and they are all recommended **[Class I, Level A]**

	For the demonstration of more distal acute branch occlusions, or for evaluation of subacute to chronic stenoses, vasospasm, and vasculitis, DSA surpasses CTA and MRA and should be used. **[Class I, Level A]**<br><br>For the detection of vascular stenoses and aneurysms, CTA and DSA are recommended **[Class I, Level A]**, whereas MRA is less accurate but can be useful **[Class IIa, Level A].**<br><br>It is important to evaluate the extracranial vasculature soon after the onset of acute cerebral ischemia to aid in the determination of the mechanism of the stroke, and thus potentially prevent a recurrence. In addition, CEA or angioplasty/stenting is occasionally performed acutely, which requires appropriate imaging **[Level B].**<br><br>TCD is useful for monitoring the development of vasospasm in large vessels at the base of the brain **[Level A]** and for determining major occlusive disease in those arteries, although CTA, MRA, and DSA are more accurate for occlusive/stenotic lesions **[Level A]**. TCD is also useful for monitoring large brain vessels in patients with sickle cell disease **[Level A]**.

*Triage categories:

- VERY HIGH risk for recurrent stroke (symptom onset within last 48 h): investigations completed as soon as possible within 24hours.
- HIGH risk for recurrent stroke (symptom onset between 48 h and two weeks): investigations ideally initiated within 24h of first contact.
- MODERATE (INCREASED) risk for recurrent stroke (symptom onset between 48 h and two weeks): investigations within two weeks of first contact.
- LOWER risk for recurrent stroke (time lapse since symptom onset greater than two weeks): investigations ideally within one month of symptom onset.

Table IV. Recommendations for ECG monitoring

Guideline	Recommendations
Powers et al. (2018) 2018 Guidelines for the Early Management of Patients With Acute Ischemic Stroke A Guideline for Healthcare Professionals From the American Heart Association/American Stroke Association	Baseline ECG assessment is recommended in patients presenting with AIS, but should not delay initiation of IV alteplase. **[Class I, Level B-NR]**
Boulanger et al. (2018) Canadian Stroke Best Practice Recommendations for Acute Stroke Management: Prehospital, Emergency Department, and Acute Inpatient Stroke Care, 6th Edition, Update 2018	Patients with suspected TIA or ischemic stroke should have a 12-lead ECG to assess cardiac rhythm and identify atrial fibrillation or flutter or evidence of structural heart disease (e.g., myocardial infarction, left ventricular hypertrophy). [**Level B]**<br><br>For patients being investigated for an acute embolic ischemic stroke or TIA, ECG monitoring for more than 24 h is recommended as part of the initial stroke work-up to detect paroxysmal atrial fibrillation in patients who would be potential candidates for anticoagulant therapy. [**Level A]**<br><br>For patients being investigated for an acute embolic ischemic stroke or TIA of undetermined source whose initial short-term ECG monitoring does not reveal atrial fibrillation but a cardioembolic mechanism is suspected, prolonged ECG monitoring for at least two weeks is recommended to improve detection of paroxysmal atrial fibrillation in selected patients aged 55 years who are not already receiving anticoagulant therapy but would be potential anticoagulant candidates. [**Level A]**<br><br>2.1.1 VERY HIGH risk for recurrent stroke (symptom onset within last 48 h) iv. An electrocardiogram (ECG) should be completed without delay. [**Level B]**
Wein et al. (2018) Canadian stroke best practice recommendations: Secondary prevention of stroke, sixth edition practice guidelines, update 2017	Patients with suspected transient ischemic attack or ischemic stroke should have a 12-lead ECG to assess cardiac rhythm and identify atrial fibrillation or flutter or evidence of structural heart disease (e.g. myocardial infarction, left ventricular hypertrophy). [**Level B]**

	For patients being investigated for an acute embolic ischemic stroke or transient ischemic attack, ECG monitoring for more than 24 hours is recommended as part of the initial stroke work-up to detect paroxysmal atrial fibrillation in patients who would be potential candidates for anticoagulant therapy. [**Level A]**<br><br>For patients being investigated for an acute embolic ischemic stroke or transient ischemic attack of undetermined source whose initial short-term ECG monitoring does not reveal atrial fibrillation but a cardioembolic mechanism is suspected, prolonged ECG monitoring for at least 2 weeks is recommended to improve detection of paroxysmal atrial fibrillation in selected patients aged 55 years who are not already receiving anticoagulant therapy but would be potential anticoagulant candidates. [**Level A]**
Joung et al. (2018) 2018 Korean guideline of atrial fibrillation management	In patients with transient ischemic attack (TIA) or ischemic stroke, screening for AF is recommended by short-term ECG recording followed by continuous ECG monitoring for at least 72 hours. **[Class I, Level B]**<br><br>In stroke patients, additional ECG monitoring by long-term noninvasive ECG monitors or implanted loop recorders should be considered to document silent atrial fibrillation. **[Class IIa, Level B,]**
Stroke Foundation/ Australian Department of Health (2017) Clinical Guidelines for Stroke Management 2017	Initial ECG monitoring should be undertaken for all patients with stroke. The duration and mode of monitoring should be guided by individual patient factors but would generally be recommended for at least the first 24 hours. **[Weak recommendation, Moderate quality of evidence]**<br><br>For patients with embolic stroke of uncertain source, longer term ECG monitoring (external or implantable) should be used**. [Strong recommendation, Moderate quality of evidence]**
Intercollegiate Stroke Working Party (2016) National Clinical Guideline for Stroke [Royal College of Physicians, UK]	People with ischaemic stroke or TIA who would be eligible for secondary prevention treatment for atrial fibrillation (anticoagulation or left atrial appendage device closure) should undergo a period of prolonged (at least 12 hours) cardiac monitoring. **[Reflected in wording]**

	People with ischaemic stroke or TIA who would be eligible for secondary prevention treatment for atrial fibrillation and in whom no other cause of stroke has been found should be considered for more prolonged ECG monitoring (24 hours or longer), particularly if they have a pattern of cerebral ischaemia on brain imaging suggestive of cardioembolism. **[Reflected in wording]**
Kirchhof et al. (2016) 2016 ESC Guidelines for the management of atrial fibrillation developed in collaboration with EACTS	In patients with TIA or ischaemic stroke, screening for AF is recommended by short-term ECG recording followed by continuous ECG monitoring for at least 72 hours. **[Class I, Level B]**<br><br>In stroke patients, additional ECG monitoring by long-term non-invasive ECG monitors or implanted loop recorders should be considered to document silent atrial fibrillation. **[Class IIa, Level B]**
Ministry of Public Health (2016) Stroke and transient ischemic attack [Qatar]	Holter monitoring:<br>• Should be performed in all patients with ischaemic stroke or TIA for 24-48 hours to identify underlying arrhythmia as a possible cause of the stroke<br>• Prolonged monitoring for up to 6 weeks (with weekly trace interpretation) will be introduced in Qatar in due course. **[Guideline Development Group Recommendation]**
Verma et al. (2014) 2014 focused update of the Canadian cardiovascular society guidelines for the management of atrial fibrillation	For patients being investigated for an acute embolic ischemic stroke or TIA, we recommend at least 24 hours of ECG monitoring to identify paroxysmal AF in potential candidates for OAC therapy. **[Strong recommendation, Moderate quality of evidence]**<br><br>For selected older patients with an acute, nonlacunar, embolic stroke of undetermined source for which AF is suspected but unproven, we suggest additional ambulatory monitoring (beyond 24 hours) for AF detection, where available, if it is likely that OAC therapy would be prescribed if prolonged AF is detected (there are currently insufficient data to indicate what the minimum AF duration should be for OAC to be instituted, and expert opinion varies widely). **[Conditional recommendation, Moderate quality of evidence]**

National Institute for Health and Care Excellence (2014) Atrial fibrillation: management (CG180) [UK]	Perform an electrocardiogram (ECG) in all people, whether symptomatic or not, in whom atrial fibrillation is suspected because an irregular pulse has been detected. **[Reflected in wording]**<br><br>In people with suspected paroxysmal atrial fibrillation undetected by standard ECG recording:<br>• use a 24-hour ambulatory ECG monitor in those with suspected asymptomatic episodes or symptomatic episodes less than 24 hours apart<br>• use an event recorder ECG in those with symptomatic episodes more than 24 hours apart **[Reflected in wording]**
Oliveira-Filho et al. (2012) Guidelines for acute ischemic stroke treatment – part I [Brazil]	Thus, it is well established the requirement, on admission, of exams, such as complete blood count, blood glucose and glycozilated haemoglobin (in cases of hyperglycemia), creatinine, urea, electrolytes, arterial blood gas analysis and coagulation, as well as electrocardiogram and cardiac enzymes, due to the common comorbidity of acute myocardial infarction. **[Grade D, Level 5]**
Bryer et al. (2010) South African guideline for management of ischaemic stroke and transient ischaemic attack 2010: A guideline from the South African Stroke Society (SASS) and the SASS Writing Committee	All acute stroke (and TIA) patients should have a 12-lead ECG. **[Class I, Level A]**<br><br>Stroke and TIA patients seen after the acute phase should have 24-hour Holter ECG monitoring when arrhythmias are suspected and no other causes of stroke are found. **[Class I, Level A]**
**Consensus documents/statements**	**Recommendations**
Gorenek et al. (2017) Device-detected subclinical atrial tachyarrhythmias: definition, implications and management—an European Heart Rhythm Association (EHRA) consensus document, endorsed by Heart RhythmSociety (HRS), Asia Pacific Heart Rhythm Society (APHRS) and Sociedad Latinoamericana de Estimulacióín Cardíaca y Electrofisiología (SOLEACE).	Outside of the research context patients with cryptogenic stroke may not receive an ILR (implantable loop recorder). **[May be used or recommended]**<br><br>Novel user-friendly external devices for AF detection have the potential to increase the yield of identifying silent AF as an aetiology for ischemic stroke. **[Recommended/indicated]**

Prasad et al. (2014) Recommendations for the Early Management of Acute Ischemic Stroke: A Consensus Statement for Healthcare Professionals from the Indian Stroke Association [India]	Cardiac monitoring is recommended to screen for atrial fibrillation and other potentially serious cardiac arrhythmias that would necessitate emergency cardiac interventions. Cardiac monitoring should be performed for at least the first 24 hours.
Summers et al. (2009) Comprehensive Overview of Nursing and Interdisciplinary Care of the Acute Ischemic Stroke Patient: A Scientific Statement From the American Heart Association	Continuous cardiac monitoring of the stroke patient should be provided for at least 24 to 48 hours after stroke to detect potential cardiac problems. **[Class I, Level B]**

Table V. Recommendations for investigations of cardiac structure

Guideline	Recommendations
Boulanger et al. (2018) Canadian Stroke Best Practice Recommendations for Acute Stroke Management: Prehospital, Emergency Department, and Acute Inpatient Stroke Care, 6th Edition, Update 2018	Echocardiography could be considered in cases where a stroke mechanism has not been identified. **[Level C]**<br><br>Echocardiography (2D or TEE) may be considered in patients where a cardiac cause of stroke is suspected, including in young adults and children who present with stroke, and when infectious endocarditis is suspected. **[Level C]**<br><br>Echocardiography, either 2D or transesophageal, should be considered for patients with suspected embolic stroke and normal neurovascular imaging [Evidence Level B] as well as no contraindications for anticoagulant therapy. This is particularly relevant for younger adults with stroke or TIA and unknown etiology. **[Level B]**
Wein et al. (2018) Canadian stroke best practice recommendations: Secondary prevention of stroke, sixth edition practice guidelines, update 2017	Echocardiography should be considered in cases where a stroke mechanism has not been identified**. [Level C]**
Stroke Foundation/ Australian Department of Health (2017) Clinical Guidelines for Stroke Management 2017	Further cardiac investigations should be performed where clarification of stroke aetiology is required after initial investigations. In patients with ischaemic stroke, echocardiography should be considered based on individual patient factors. Transoesophageal echocardiography is more sensitive for suspected valvular, left atrial and aortic arch pathology. **[Weak recommendation, Low quality of evidence]**
Saric et al. (2016) Guidelines for the Use of Echocardiography in the Evaluation of a Cardiac Source of Embolism [American Society of Echocardiography]	[No formal grading system]<br><br>Appropriate Use: TEE<br>• As initial or supplemental test for evaluation for cardiovascular source of embolus with no identified noncardiac source<br><br>Uncertain Indication for Use: TEE

	• Evaluation for cardiovascular source of embolus with a previously identified noncardiac source<br><br>Inappropriate Use: TEE<br>• Evaluation for cardiovascular source of embolus with a known cardiac source in which TEE would not change management<br><br>Echocardiography Recommended<br>• Echocardiography should be considered in all patients with suspected cardiac sources of embolism, especially in patients for whom clinical therapeutic decisions (such as anticoagulation or cardioversion) will depend on echocardiographic findings.<br><br>TTE versus TEE<br>• TEE is not indicated when transthoracic echocardiographic findings are diagnostic for a cardiac source of embolism.<br>• TTE may be unnecessary when TEE is already planned (e.g., for evaluation of intracardiac masses, prosthetic valves, and thoracic aorta or when TEE is used to guide a percutaneous procedure related to cardiac source of embolism).<br><br>Alternative Imaging Recommended<br>• Computed tomographic and magnetic resonance neuroimaging is essential in the evaluation of patients with neurologic symptoms attributable to a cardiac source of emboli.<br>• CT, MRI, or other radiologic imaging of the heart and the great vessels may be useful in selected patients with cardiac sources of embolism.<br><br>Echocardiography Recommended<br>• TTE is recommended for the evaluation of a right-to-left shunt and atrial septal anatomy in a patient who presents with cryptogenic stroke, especially in the setting of elevated right atrial pressure with documented PE or deep venous thrombosis of lower extremities or pelvic veins.

Intercollegiate Stroke Working Party (2016) National Clinical Guideline for Stroke [Royal College of Physicians, UK]	People with stroke or TIA should be investigated with transthoracic echocardiography if the detection of a structural cardiac abnormality would prompt a change of management and if they have:<br>• clinical or ECG findings suggestive of structural cardiac disease that would require assessment in its own right, or<br>• unexplained stroke or TIA, especially if other brain imaging features suggestive of cardioembolism are present. **[Reflected in wording]**
Ministry of Public Health (2016) Stroke and transient ischemic attack [Qatar]	Echocardiogram:<br>• Patients with ischaemic stroke or TIA should not routinely undergo transthoracic echocardiogram in the acute setting.<br>• In younger patients, transoesophageal echocardiogram may be considered to identify underlying cardiac pathology. **[not provided]**
**Consensus documents/statements**	**Recommendations**
Pepi et al. (2010) Recommendations for echocardiography use in the diagnosis and management of cardiac sources of embolism [European Association of Echocardiography (EAE)]	[No formal grading system]<br><br>TTE and TOE are recommended when symptoms potentially due to a suspected cardiac aetiology including syncope, TIA, and cerebrovascular events are present<br><br>TOE is traditionally the gold standard for the detection of PFO, however in the presence of good image quality, transthoracic echo is sufficient to detect the presence of a PFO. Performance of a valid Valsalve manoeuvre or strong cough must be ensured with both methods<br><br>In patients with stroke, the use of suprasternal TTE may help to identify arch atheromas. TOE may be indicated when image quality is inadequate to reliably rule out atheromas or define plaque characteristics so that specific therapies can be considered<br><br>In patients with peripheral embolism, when TTE fails to identify the source of embolism, TOE is the technique of choice for the detection of mobile lesions superimposed on aortic atheromas or to rule out the presence of large, mobile, or pedunculated thrombi

	Echocardiography is recommended in patients with known MVP, MAC, or aortic stenosis and an embolic event

Table VI. Recommendations for laboratory investigations

Guideline	Recommendations
Powers et al. (2018) 2018 Guidelines for the Early Management of Patients With Acute Ischemic Stroke A Guideline for Healthcare Professionals From the American Heart Association/American Stroke Association	Baseline troponin assessment is recommended in patients presenting with AIS, but should not delay initiation of IV alteplase. **[Class I, Level B-NR]**
Boulanger et al. (2018) Canadian Stroke Best Practice Recommendations for Acute Stroke Management: Prehospital, Emergency Department, and Acute Inpatient Stroke Care, 6th Edition, Update 2018	The following laboratory investigations should be routinely considered for patients with TIA or nondisabling ischemic stroke as part of the initial evaluation:<br>a. Initial bloodwork: hematology (complete blood count), electrolytes, coagulation (aPTT, INR), renal function (creatinine, e-glomerular filtration rate), random glucose and troponin. **[Level C]**<br>b. Subsequent laboratory tests may be considered during patient encounter or as an outpatient, including a lipid profile (fasting or non-fasting); and screening for diabetes with either a glycated hemoglobin (HbA1c) or 75 g oral glucose tolerance test. **[Level C]**
Wein et al. (2018) Canadian stroke best practice recommendations: Secondary prevention of stroke, sixth edition practice guidelines, update 2017	The following laboratory investigations should be routinely considered for patients with transient ischemic attack or nondisabling ischemic stroke as part of the initial evaluation:<br>a. Initial bloodwork: hematology (complete blood count), electrolytes, coagulation (aPTT, INR), renal function (creatinine, e-glomerular filtration rate), random glucose or hemoglobin A1c, and troponin. **[Level C]**<br>b. Subsequent laboratory tests may be considered during patient encounter or as an outpatient, including a lipid profile (fasting or nonfasting); and, screening for diabetes with either a fasting plasma glucose, or 2-hour plasma glucose, or glycated hemoglobin (A1C), or 75 g oral glucose tolerance test. **[Level C]**
Stroke Foundation/ Australian Department of Health (2017) Clinical Guidelines for Stroke Management 2017	Not addressed as a formal recommendation but it is suggested that “Routine investigations should include full blood count, electrolytes, erythrocyte sedimentation rate, C-reactive protein, renal function, cholesterol and glucose levels, although direct evidence is lacking for each of these investigations.”

Oliveira-Filho et al. (2012) Guidelines for acute ischemic stroke treatment - part I [Brazil]	Thus, it is well established the requirement, on admission, of exams, such as complete blood count, blood glucose and glycozilated hemoglobin (in cases of hyperglycemia), creatinine, urea, electrolytes, arterial blood gas analysis and coagulation, as well as electrocardiogram and cardiac enzymes, due to the common comorbidity of acute myocardial infarction.**[Grade D, Level 5]**<br><br>Exams to be requested in the sub-acute phase: lipid profile, serology for Chagas' disease and syphilis, and, in young patients, in addition to the ones already mentioned, evaluation of autoimmune diseases, arteritis, homocysteine levels, AVM research, coagulopathy and genetic profile for thrombophylia. **[Grade D, Level 5]**
Bryer et al. (2010) South African guideline for management of ischaemic stroke and transient ischaemic attack 2010: A guideline from the South African Stroke Society (SASS) and the SASS Writing Committee	In patients with acute stroke and TIA, early clinical evaluation, including physiological parameters and routine blood tests, is recommended. **[Class I, Level A]**
**Consensus documents/statements**	**Recommendations**
Summers et al. (2009) Comprehensive Overview of Nursing and Interdisciplinary Care of the Acute Ischemic Stroke Patient: A Scientific Statement From the American Heart Association	In the ED setting, laboratory tests should be obtained and processed rapidly to facilitate rapid assessment of the stroke patient, especially one who is a candidate for rtPA. At a minimum, the following tests should be performed: CBC, including platelets, blood chemistries, and coagulation studies (PT, aPTT, and INR). **[Class I, Level A]**

Table VII. Recommendations for other investigations

Guideline	Recommendations
Powers et al. (2018) 2018 Guidelines for the Early Management of Patients With Acute Ischemic Stroke A Guideline for Healthcare Professionals From the American Heart Association/American Stroke Association	Usefulness of chest radiographs in the hyperacute stroke setting in the absence of evidence of acute pulmonary, cardiac, or pulmonary vascular disease is unclear. If obtained, they should not unnecessarily delay administration of IV alteplase. **[Class IIB, Level B-NR]**
Boulanger et al. (2018) Canadian Stroke Best Practice Recommendations for Acute Stroke Management: Prehospital, Emergency Department, and Acute Inpatient Stroke Care, 6th Edition, Update 2018	A chest x-ray should be completed when the patient has evidence of acute heart disease or pulmonary disease. **[Level B]**
Intercollegiate Stroke Working Party (2016) National Clinical Guideline for Stroke [Royal College of Physicians, UK]	People with ischaemic stroke or TIA in whom other conditions such as atrial fibrillation and large or small vessel atherosclerotic disease have been excluded should be investigated for antiphospholipid syndrome (with IgG and IgM anticardiolipin ELISA and lupus anticoagulant), particularly if the person:<br>• is under 50 years of age;<br>• has any autoimmune rheumatic disease, particularly systemic lupus erythematosus;<br>• has a history of one or more venous thromboses;<br>• has a history of recurrent first trimester pregnancy loss or at least one late pregnancy loss (second or third trimester). **[reflected in wording]**<br><br>Young people with stroke or TIA should be investigated for Fabry disease if they have suggestive clinical features such as acroparesthesias, angiokeratomas, sweating abnormalities, corneal opacities, unexplained renal insufficiency or a family history suggesting the condition. **[reflected in wording]**
Ministry of Public Health (2016) Stroke and transient ischemic attack [Qatar]	Screening for thrombophilic state:<br>• May be appropriate for younger patients (age <50 years) with no other risk factors identified for ischaemic stroke or TIA. **[not provided]**

National Institute for Health and Care Excellence (2014) Atrial fibrillation: management (CG180) [UK]	Perform manual pulse palpation to assess for the presence of an irregular pulse that may indicate underlying atrial fibrillation in people presenting with any of the following:<br>• breathlessness/dyspnoea<br>• palpitations<br>• syncope/dizziness<br>• chest discomfort<br>• stroke/transient ischaemic attack. **[reflected in wording]**

Table VIII. Commentary in the guidelines/statements relating to when a stroke should be classified as cryptogenic

Guideline	Relevant text
National Institute for Health and Care Excellence (2019) Stroke and transient ischaemic attack in over 16s: diagnosis and initial management (NG128) [UK]	No relevant text
Powers et al. (2018) 2018 Guidelines for the Early Management of Patients With Acute Ischemic Stroke A Guideline for Healthcare Professionals From the American Heart Association/American Stroke Association	No relevant text
Boulanger et al. (2018) Canadian Stroke Best Practice Recommendations for Acute Stroke Management: Prehospital, Emergency Department, and Acute Inpatient Stroke Care, 6th Edition, Update 2018	The guideline does not refer to cryptogenic stroke but does make reference to embolic stroke of undetermined source (ESUS). Recommendations relate to prolonged ECG monitoring (at least 2 weeks) to detect paroxysmal atrial fibrillation. No further guidance or recommendations are provided about continued evaluation in instances where underlying etiology has not been determined.
Wein et al. (2018) Canadian stroke best practice recommendations: Secondary prevention of stroke, sixth edition practice guidelines, update 2017	The guideline does makes a single reference to cryptogenic stroke when describing the results of the RESPECT trial. As per Boulanger et al. (2018) the guideline also makes reference to embolic stroke of undetermined source (ESUS). Recommendations relate to prolonged ECG monitoring (at least 2 weeks) to detect paroxysmal atrial fibrillation. No further guidance or recommendations are provided about continued evaluation in instances where underlying etiology has not been determined.

Joung et al. (2018) 2018 Korean guideline of atrial fibrillation management	The authors cite the TOAST classification when providing the following definition of cryptogenic stroke:<br><br>Cryptogenic stroke defined as the cause of ischemic stroke remains uncertain despite a complete diagnostic evaluation. (p.1040)<br><br>Cryptogenic stroke is discussed in the context of prolonged ECG monitoring and the authors recommend that all stroke patients undergo prolonged ECG monitoring (length of time not defined) to document silent atrial fibrillation.
Stroke Foundation/Australian Department of Health (2017) Clinical Guidelines for Stroke Management 2017	This guideline uses the ESUS definition:<br><br>Embolic stroke of uncertain source (ESUS) is a relatively recently defined subgroup of what has previously been called "cryptogenic stroke". It aims to define a group at higher risk of occult paroxysmal atrial fibrillation and comprises a non-lacunar infarct in the absence of significant proximal vessel disease, a normal echocardiogram and at least 24 hours of unremarkable ECG monitoring. (Chapter 2, p.47)<br><br>For this subgroup of patients longer term ECG monitoring is recommended (length of time not defined in light of lack of evidence). No further guidance or recommendations are provided about continued evaluation in instances where underlying etiology has not been determined.
Saric et al. (2016) Guidelines for the Use of Echocardiography in the Evaluation of a Cardiac Source of Embolism [American Society of Echocardiography]	This guideline cites the TOAST classification and defines cryptogenic stroke as “a stroke of unknown etiology, despite extensive evaluation”. The guideline discusses in detail the role of echocardiography in evaluating embolic strokes and cryptogenic strokes but no guidance or recommendations are provided about continued evaluation in instances where underlying etiology is not established through these investigations.

Intercollegiate Stroke Working Party (2016) National Clinical Guideline for Stroke [Royal College of Physicians, UK]	The guideline makes reference to cryptogenic or unexplained stroke in the context of describing the potential for paroxysmal atrial fibrillation and patent foramen ovale to act as underlying causes of stroke. Additionally, the guideline makes reference to stroke of undetermined etiology when detailing the potential value of transthoracic echocardiography for individuals who would be candidates for anticoagulation. No further guidance or recommendations are provided about continued evaluation in instances where underlying etiology has not been determined.
Kirchhof et al. (2016) 2016 ESC Guidelines for the management of atrial fibrillation developed in collaboration with EACTS	This guideline cites the TOAST classification and defines cryptogenic stroke as “a stroke in which the cause could not be identified after extensive investigations” (p.1621). As this guideline relates to atrial fibrillation, cryptogenic stroke was discussed in the context of prolonged monitoring to identify silent AF.
Ministry of Public Health (2016) Stroke and transient ischemic attack [Qatar]	This guideline cites the TOAST criteria where cryptogenic stroke or stroke of undetermined cause includes instances where there has been incomplete evaluation for cause; where diagnostic studies were negative; and where ≥2 conflicting causes have been identified. A general list of investigations is provided but no specific guidance or recommendations are provided related to instances where underlying etiology has not been determined.
Verma et al. (2014) 2014 focused update of the Canadian cardiovascular society guidelines for the management of atrial fibrillation	As this guideline relates to atrial fibrillation, cryptogenic stroke and embolic stroke of undetermined source are discussed in the context of short duration ECG monitoring which is said to likely result in missed detection of AF cases. The guideline makes reference to the five TOAST categories and also provides the following definition:<br><br>As many as 1 in 4 ischemic strokes, with no cause identified after the usual post-stroke diagnostic evaluation, is classified as ‘cryptogenic stroke’ or ‘embolic stroke of undetermined source.’ (p.1118)

National Institute for Health and Care Excellence (2014) Atrial fibrillation: management (CG180) [UK]	No relevant text
Oliveira-Filho et al. (2012) Guidelines for acute ischemic stroke treatment - part I [Brazil]	No relevant text
Ministry of Health Malaysia, Academy of Medicine Malaysia, Malaysian Society of Neurosciences (2010) Management of Ischaemic Stroke (2nd Edition)	This guideline does not make explicit reference to cryptogenic stroke or stroke of unknown source but provides details on investigations in different stroke subgroups. No detail is provided about the extent to which cases should be investigated to identifying underlying cause.
Bryer et al. (2010) South African guideline for management of ischaemic stroke and transient ischaemic attack 2010: A guideline from the South African Stroke Society (SASS) and the SASS Writing Committee	Cryptogenic stroke is mentioned in the context of PFO closure. The guideline also mentions stroke of unknown cause in the context of describing the levels facilities to which different types of stroke should be transferred. A list of investigations is provided but no specific guidance or recommendations are provided about the extent to which cases should be investigated if initial investigations do not identify underlying cause.
Ministry of Health (2009) Stroke and transient ischaemic attacks. Assessment, investigation, immediate management and secondary prevention. [Singapore]	No relevant text
**Consensus documents/statements**	**Relevant text**
Gorenek et al. (2017) Device-detected subclinical atrial tachyarrhythmias: definition, implications and management—an European Heart Rhythm Association (EHRA) consensus document, endorsed by Heart RhythmSociety (HRS), Asia Pacific Heart Rhythm Society (APHRS) and Sociedad Latinoamericana de Estimulacióín Cardíaca y Electrofisiología (SOLEACE).	The authors provide the following definition of cryptogenic stroke:<br>Cryptogenic stroke is defined as an embolic (defined by brain imaging characteristics) cerebrovascular infarct for which no underlying cause can be identified after full cardiovascular evaluation including exclusion of intracranial shunts and carotid/vertebral arterial disease by appropriate imaging studies, and ‘thrombogenic’ arrhythmias such as AF, atrial flutter

	and, more recently, high frequency atrial premature beats by continuous electrocardiographic monitoring. (p.1570)<br><br>In light of the focus of the document, cryptogenic stroke is discussed in terms of the existing evidence base around monitoring for atrial fibrillation.<br><br>The consensus document also mentions ESUS and outlines that "There is much similarity between the phenotype of cryptogenic stroke (embolic stroke of uncertain source [ESUS]) and AF-related stroke." (p.1571)
Prasad et al. (2014) Recommendations for the Early Management of Acute Ischemic Stroke: A Consensus Statement for Healthcare Professionals from the Indian Stroke Association [India]	This guideline does not make explicit reference to cryptogenic stroke or stroke of unknown source but provides a list of investigations which could be used to identify underlying cause. No detail is provided about the extent to which cases should be investigated to identifying underly cause.
Wintermark et al. (2013) Imaging recommendations for acute stroke and transient ischemic attack patients: a joint statement by the American Society of Neuroradiology, the American College of Radiology and the Society of NeuroInterventional Surgery [American Society of Neuroradiology]	No relevant text
Pepi et al. (2010) Recommendations for echocardiography use in the diagnosis and management of cardiac sources of embolism [European Association of Echocardiography (EAE)]	The guideline employs the TOAST classification system to differentiate stroke subtypes and define cryptogenic stroke.<br><br>The TOAST criteria are the most frequently used classification of stroke in epidemiological or genetic studies and refer to (i) large-artery atherosclerosis (artery-to-artery embolus, large artery atherothrombosis), (ii) cardiac embolism, (iii) cerebral small artery occlusion (lacunar stroke), (iv) stroke of another determined aetiology (rare aetiologies), and (v) stroke of undetermined aetiology.[14] The latter category refers to cryptogenic strokes, but is also chosen if two or more causes of stroke can be identified in the same patient, or—even more questionably—if the patient has a negative or

	incomplete evaluation. Categories 2 and 5 are of particular interest for echocardiography. (p.463)<br><br>As the focus of the recommendations are around diagnosing cardiac sources of embolism, no further information is provided about investigating other underlying causes prior to classifying a stroke as cryptogenic.
Irimia et al. (2011) Use of imaging in cerebrovascular disease [European Federation of Neurological Societies]	No reference made to cryptogenic stroke or stroke of unknown source, guidance relates to the role of imaging in establishing underlying cause but no detail is provided about the extent to which cases should be investigated to identify underlying cause.
Summers et al. (2009) Comprehensive Overview of Nursing and Interdisciplinary Care of the Acute Ischemic Stroke Patient: A Scientific Statement From the American Heart Association	Cryptogenic stroke briefly discussed in the context of detailing stroke subtypes. No detail is provided about the extent to which cases should be investigated to identify underlying cause.
Latchaw et al. (2009) Recommendations for Imaging of Acute Ischemic Stroke: A Scientific Statement From the American Heart Association	No relevant text
